# Canine Intestinal Organoids as a Novel In Vitro Model of Intestinal Drug Permeability: A Proof-of-Concept Study

**DOI:** 10.3390/cells12091269

**Published:** 2023-04-27

**Authors:** Dipak Kumar Sahoo, Marilyn N. Martinez, Kimberly Dao, Vojtech Gabriel, Christopher Zdyrski, Albert E. Jergens, Todd Atherly, Chelsea A. Iennarella-Servantez, Laura E. Burns, Dwayne Schrunk, Donna A. Volpe, Karin Allenspach, Jonathan P. Mochel

**Affiliations:** 1 Department of Veterinary Clinical Sciences, Iowa State University, Ames, IA 50011, USA; dipaksahoo11@gmail.com (D.K.S.); allek@iastate.edu (K.A.); 2 Office of New Animal Drug Evaluation, Center for Veterinary Medicine, Food and Drug Administration, Rockville, MD 20852, USA; 3 3D Health Solutions, Iowa State University, Ames, IA 50011, USA; 4 Department of Biomedical Sciences, SMART Pharmacology, Iowa State University, Ames, IA 50011, USA; 5 Veterinary Diagnostic Laboratory, Iowa State University, Ames, IA 50011, USA; 6 Division of Applied Regulatory Science, Office of Clinical Pharmacology, Center for Drug Evaluation and Research, Food and Drug Administration, Silver Spring, MD 20852, USA

**Keywords:** canine, 3D organoid, permeability, Caco-2, colon

## Abstract

A key component of efforts to identify the biological and drug-specific aspects contributing to therapeutic failure or unexpected exposure-associated toxicity is the study of drug–intestinal barrier interactions. While methods supporting such assessments are widely described for human therapeutics, relatively little information is available for similar evaluations in support of veterinary pharmaceuticals. There is, therefore, a critical need to develop novel approaches for evaluating drug–gut interactions in veterinary medicine. Three-dimensional (3D) organoids can address these difficulties in a reasonably affordable system that circumvents the need for more invasive in vivo assays in live animals. However, a first step in developing such systems is understanding organoid interactions in a 2D monolayer. Given the importance of orally administered medications for meeting the therapeutic need of companion animals, we demonstrate growth conditions under which canine-colonoid-derived intestinal epithelial cells survive, mature, and differentiate into confluent cell systems with high monolayer integrity. We further examine the applicability of this canine-colonoid-derived 2D model to assess the permeability of three structurally diverse, passively absorbed β-blockers (e.g., propranolol, metoprolol, and atenolol). Both the absorptive and secretive apparent permeability (*P_app_*) of these drugs at two different pH conditions were evaluated in canine-colonoid-derived monolayers and compared with that of Caco-2 cells. This proof-of-concept study provides promising preliminary results with regard to the utility of canine-derived organoid monolayers for species-specific assessments of therapeutic drug passive permeability.

## 1. Introduction

**Figure 1:**
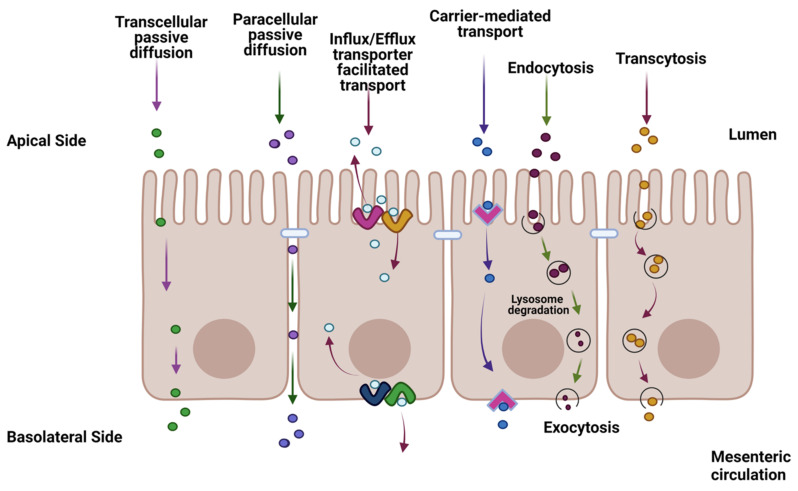
Summary diagram of the transport of a therapeutic drug through the intestine facilitated by transcellular transport, carrier-mediated (CM), and passive diffusion pathways such as passive lipoidal diffusion, CM influx, CM efflux, paracellular diffusion, endocytosis, and transcytosis. The figure was produced with BioRender (www.biorender.com; accessed on 11 January 2023).

Oral dosing is the most-common route of administration for human therapeutics, accounting for over 90% of all drug formulations [1]. For systemically active pharmaceuticals, orally delivered dosage forms must be absorbed by the gastrointestinal (GI) tract to exert their clinical effects at the target site. Disintegration, dissolution, and permeation are three key processes involved in the GI absorption of solid dosage forms [2,3]. Solubilized molecules must pass through the single layer of enterocytes lining the gut lumen to be absorbed into the portal circulation. Thus, the intestinal epithelium serves both as an absorptive surface and an absorption barrier to the systemic entry of therapeutic drugs. The barrier functions of the enterocytes include metabolizing enzymes within the cells, membrane efflux transporters, and tight junctions (TJs), the latter consisting of extracellular folds of the transmembrane proteins and multiprotein junctional complexes that form pore-like structures constraining movement across the intercellular spaces [4] (Figure 1).

**Figure 2:**
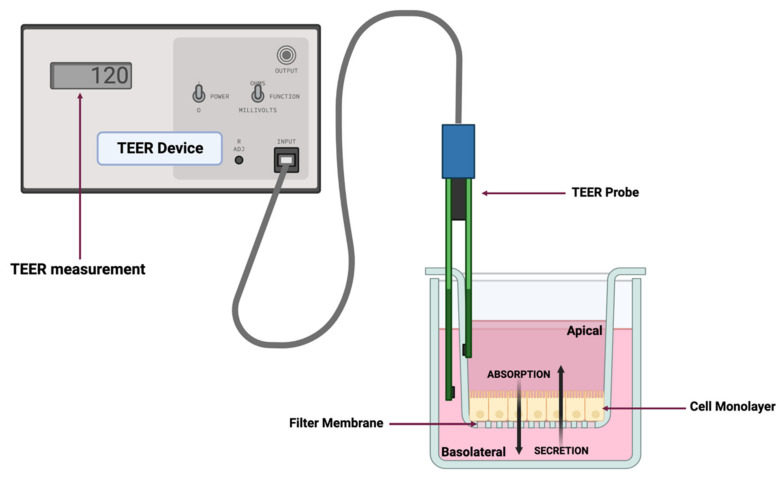
Representative diagram of a dual-chamber culture apparatus showing a cell monolayer grown on a filter membrane with apical and basolateral chambers and validation of monolayer integrity by transepithelial electrical resistance (TEER) measurement. Absorption is measured by adding drug to the apical chamber and measuring its appearance in the basolateral chamber over time. Conversely, secretion (efflux) is measured by adding drug to the basolateral chamber and measuring its appearance in the apical chamber over time. The figure was produced with BioRender (www.biorender.com; accessed on 11 January 2023).

Movement of a drug (rate of flux) in a diffusion cell system from a donor compartment to a receiver compartment can be used to obtain a mathematical estimate of the apparent permeability (*P_app_*) (Figure 2). The *P_app_* estimate, which is a function of both passive and active transport mechanisms, can be derived using a variety of in vitro systems [5]. Excluding the possibility of drug metabolism within the enterocyte, drug absorption can be evaluated within monolayer cell systems such as those associated with the parallel artificial membrane permeability assay (PAMPA), which typically reflects passive permeability only [6], the human colon adenocarcinoma (Caco-2) cell line [7,8,9,10], and more recently, intestinal organoids [11].

Mathematically, the in vitro *P_app_* can be used to estimate in vivo effective permeability (*P_eff_*) by incorporating information on the fraction of drug molecules existing in their neutral form at a given pH, the surface area for absorption, the fraction of unbound drug in the unstirred boundary layer, and the drug permeability across the unstirred boundary layer [12]. The determination of a drug *P_app_* early within the drug product development process is critical for predicting possible challenges with oral bioavailability and setting up corresponding formulation strategies to circumvent these issues. Cell culture systems, e.g., Caco-2, are frequently employed to determine oral drug permeability and are currently considered the gold standard for estimating in vitro intestinal permeability and oral absorption of candidate therapeutics [13]. The Caco-2 cell line forms monolayers that both morphologically and functionally resemble the small intestinal (absorptive) enterocytes [14].

One of the reasons for the extensive use of the Caco-2 assay lies in the versatility of the cell line, allowing for the study of passive diffusion processes, active drug transport, and pre-systemic drug metabolism [15]. Caco-2 cells spontaneously differentiate into mature small intestinal enterocytes that express morphological (polarized columnar epithelium) and functional features such as intercellular TJs, efflux (e.g., P-glycoprotein (P-gp) and a member of the multidrug-resistance-associated protein (MRP) family) and influx (e.g., the organic anion transporting protein (OATP)) transporters and the presence of enzymes such as the cytochrome P450 (CYP) isoenzymes [15,16,17]. While Caco-2 cells are widely used in the pharmaceutical industry and are accepted by regulatory agencies to predict human intestinal drug permeability, caution should be exercised when extrapolating data from these in vitro models to in vivo physiology [18,19]. More specifically, in addition to the issues outlined above, there have been discrepancies in the expression of influx transporters in Caco-2 cells between studies conducted in different laboratories [20,21,22,23,24]. Additionally, one disadvantage of Caco-2 cells is that they only represent one cell type from the epithelial layer of the small intestine [25]. Specifically, the absence of goblet cells, which are responsible for mucus production, makes it impossible to evaluate mucus–drug interactions [26]. The presence of mucus from goblet cells represents a physiological barrier that drugs cross to enter the intestinal enterocytes. It should also be noted that Caco-2 cultures lack the expression of numerous key nuclear receptors normally found in the intestine, including the pregnane X receptor (PXR), steroid X receptor (SXR), and constitutive androstane receptor (CAR) [27]. As a result, Caco-2 cultures are unable to simulate the induction of drug transporters and enzymes by certain drugs (e.g., rifampin) that interact with these receptors [28].

Research in biomedical science, disease modeling, and personalized medicine has advanced since Sato et al. (2009) first reported the in vitro culture of intestinal organoids [19,29,30,31,32,33,34,35,36,37,38,39]. Three-dimensional (3D) organoids are derived from leucine-rich repeat containing G-protein-coupled receptor 5 (Lgr5)-positive adult stem cells [35,38,39]. Their 3D structure enables organoids to morphologically, physiologically, and structurally mimic endogenous epithelia. Therefore, these systems provide an opportunity to evaluate the transport and intestinal metabolism of administered oral therapeutic drugs [19]. Although human-adult-stem-cell-derived intestinal organoids are now used as intestinal permeability models [40,41], canine organoids present a promising alternative to human organoids due to ethical constraints related to research on human stem cells [42] and the lack of availability of large human organoid bioarchives. Canine intestinal organoids can also serve as a platform for appreciating potential interspecies differences in drug absorption (e.g., human preclinical species or when extrapolating human absorption data to the dog) and for predicting absorption challenges that can occur for canine-targeted oral therapeutics.

Canine-specific permeability tools are needed for assessing the unique absorption challenges associated with the canine GI tract. In this regard, although Caco-2 cells are commonly used to evaluate human drug permeability, their predictive performances in modeling *P_app_* in dogs remain to be demonstrated. The use of canine intestinal organoids in permeability studies may be more accurate in predicting canine, not human, intestinal permeability and metabolism of small pharmacological molecules as compared to that derived using Caco-2 or Madin–Darby canine kidney (MDCK) cells, with a potential to avoid the ethical and financial constraints associated with the use of live animal models. Moreover, the possibility of re-using organoids preserved in a biorepository further supports the 3R “Replacement, Reduction, and Refinement” initiative.

The potential utility of organoid systems is gaining recognition within the pharmaceutical sciences, with examples being applied to exploring drug screening, cancer therapies, gene therapy, and therapies for a host of other kinds of diseases [43,44,45]. Examples of cultured organoid systems include human kidney tubuloids [46], dog prostate cancer organoids [47], and human bladder cancer organoids [29,48,49]. Although still early in its development, with many questions and challenges yet to be addressed, there are recent efforts to improve the ability of the 3D organoid to recapitulate in vivo organ physiology through the development of the organ-on-a-chip technology [50]. Microfluidic organ-on-chips [50] provide a constant flow of media through the cell culture, while various other physiologically important parameters, such as oxygen saturation and shear stress, can be manipulated and mimicked more closely for physiological representation. However, this technology is still relatively new and bears a number of limitations, such as significant batch-to-batch variability, causing a wide range of results for the same parameters [51,52]. Furthermore, due to the complexity of the microfluidic system, experiments involving organs-on-a-chip require additional technical skill sets, thereby limiting its broader use in preclinical research [51,52].

Whether or not influx and efflux transporters are involved in the transmembrane movements of a drug candidate, it is essential that a model system does not introduce a bias by failure to adequately control passive transport. Accordingly, the first step in characterizing the function of cell monolayers is to assess their passive permeability. Furthermore, the predictive performances of the novel canine colonoid model need to be compared with those of the current gold-standard Caco-2 cell monolayer to identify possible interspecies differences in drug passive permeability. To meet this objective, the study presented herein used model drugs with well-characterized transcellular absorption (propranolol and metoprolol) and paracellular diffusion (atenolol) attributes. Since this was a proof-of-concept study, this choice was motivated by the known in vivo and in vitro permeability of these drugs. Metoprolol and propranolol are human BCS Class I (high solubility, high permeability) drugs [53], while atenolol is a BCS Class III drug (low permeability, high solubility) [54].

In this study, we thoroughly validated the integrity of canine monolayers and their functionality by transepithelial electrical resistance (TEER) measurement and the FITC-dextran assay. We also report expression data for key intestinal epithelial cell markers, tight and adherens junction proteins, transporters, and cytochrome P450 (CYP) enzymes. Finally, both the absorptive (apical to basolateral, AP→BL) and secretive (basolateral to apical, BL→AP) *P_app_* estimates of these model drugs were evaluated in Caco-2 cell and canine-colonoid-derived monolayers under various pH conditions, intended to reflect the in vivo intestinal physiology. Our working hypothesis was that the passive permeability of the two transcellularly absorbed molecules would be similar across the two cell systems, but that differences may be observed with atenolol [55]. It is noteworthily that this first proof-of-concept study focused on the number of conditions being explored rather than replicates per condition, with the option of expanding the number of replicates for studying a particular condition as needed.

## 2. Materials and Methods

### 2.1. Materials

Caco-2 cells (Passage #47) and FITC-dextran were purchased from Millipore Sigma (Sigma-Aldrich Inc, St. Louis, MO, USA). Propranolol hydrochloride, metoprolol tartrate, and atenolol were obtained from Tocris Bioscience (Bio-Techne Corporation, Minneapolis, MN, USA). Corning^®^ Transwell™ 24-well plates with permeable polyester membrane inserts (6.5 mm diameter and 0.4 μm pore size), GIBCO™ TrypLE™ express enzyme (1X, no phenol red), trypan blue solution (0.4%), Corning™ Matrigel^®^ growth factor reduced (GFR) basement membrane matrix (phenol-red-free, LDEV-free) for organoid culture, Fetal bovine serum (FBS), and GIBCO Hank’s balanced salt solution (HBSS) were purchased from Thermo Fisher Scientific (Bedford, MA, USA).

### 2.2. Caco-2 Cell Culture and Maintenance

Caco-2 cells (Passage 29) were cultured and maintained according to the methods previously described by Volpe et al. [7,56]. After rapid thawing in a 37 °C water bath, Caco-2 cells were transferred to a centrifuge tube containing 2.0 mL Dulbecco’s modified Eagle medium/Ham’s F-12 (GIBCO™ Advanced DMEM/F-12, Thermo Fisher Scientific) supplemented with 10% FBS (CCCM) and were centrifuged at 100× *g* for 5 min at 4 °C. The cell pellet was resuspended with 10 mL of Caco-2 cell culture medium (CCCM) and cultured in a cell culture flask at 37 °C in a humidified O_2_/CO_2_ incubator (Panasonic, PHC Corporation of North America, Wood Dale, IL, USA) in the presence of air supplemented with 5% CO_2_. After 24 h, CCCM media were supplemented with 100 U/mL penicillin and 100 µg/mL streptomycin (Pen-Strep). During subculture, CCCM media with Pen-Strep were replaced thrice weekly until cells attained 80% confluence.

For the subculture (passaging), Caco-2 cells at approximately 80% confluence were rinsed initially to remove all traces of trypsin inhibitor and then treated with 0.25% (*w*/*v*) trypsin and 0.53 mM EDTA solution (Thermo Fisher Scientific) at 37 °C for 10 min. The cell culture flask was observed frequently under an inverted microscope (DMi1, Leica Microsystems, Wetzlar, Germany) to monitor trypsinization progress. Over-trypsinization was avoided as it can damage cells and induce clumping. After approximately 70–80% of the cells had been detached from the surface, 3 mL of pre-warmed CCCM media was added to the cell culture flask, and cells were separated from clumps using repeated pipetting. Caco-2 cells were sub-cultured in new flasks with a medium at a ratio of 1:6 to 1:10.

### 2.3. Caco-2 Two-Dimensional Monolayer Preparation and Maintenance

Once Caco-2 cells were at least 80% confluent, epithelial cells were passaged, as described in the previous section. The harvested Caco-2 cell suspension was then filtered through a sterile cell strainer (40 µm nylon mesh, Thermo Fisher Scientific) to obtain a single-cell suspension. Caco-2 cell viability and concentration were determined by a trypan blue exclusion test [57,58]. Single Caco-2 cells (passages 50–55) were seeded at a density of 75,000 cells/cm^2^ in the apical chamber of the insert and cultured in CCCM media. The culture medium in both apical and basolateral chambers was changed every other day. In the transport studies, the monolayers were utilized 18 to 20 days after plating. Transepithelial electrical resistance (TEER) was measured every other day (described later in Section 2.6).

### 2.4. Maintenance of 3D Colonoids

The collection and analysis of canine colon biopsy samples were approved by the Iowa State University (ISU) Institutional Animal Care and Use Committee (IACUC) (IACUC-22-050). All methods were performed in accordance with the relevant guidelines and regulations of IACUC as required by U.S. federal regulations. The study is reported in accordance with the ARRIVE guidelines (https://arriveguidelines.org; accessed on 4 May 2020). The tissue used for generating the canine colonoids was obtained from a healthy male, 18-month-old dog, and colonoids were cultured and maintained according to methods previously described by our laboratory [32,35,36]. Following sample collection and upon recovery from anesthesia, the dog was returned to the colony.

**Figure 3:**
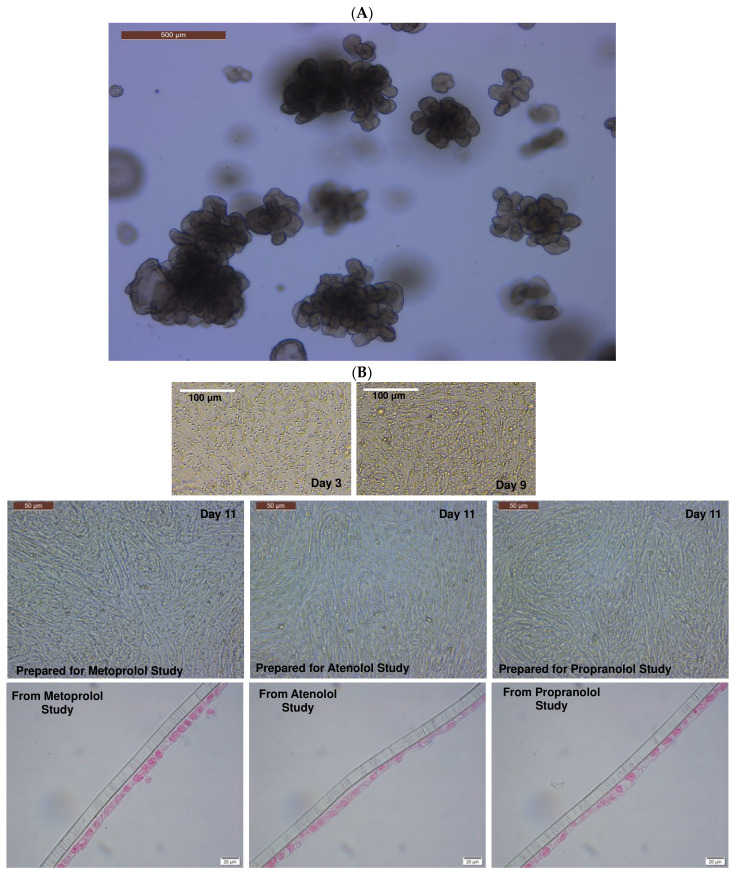
Formation of the canine-colonoid-derived monolayer. (**A**) Representative phase-contrast micrograph of a differentiated colonoid on Day 5 used for monolayer preparation. Scale bar, 500 μm. (**B**) Representative phase-contrast micrographs of colonoid-derived monolayers on Days 3, 9, and 11 and representative micrographs of eosin-stained colonoid-derived monolayers on Day 11. Scale bar, as indicated.

In brief, colonic crypts containing primary adult intestinal stem cells (ISCs) were isolated using the cold EDTA chelation method [35]. Complete medium with ISC growth factors (CMGF+) containing advanced DMEM/F12 (GIBCO) supplemented with 1:100 GlutaMAX™ supplement (Fisher), 200 mM HEPES (Fisher), and 100 μg/mL Primocin^®^ antibiotics (InvivoGen, San Diego, CA, USA), 1X B-27™ (Fisher), 1X N-2 supplement (GIBCO™), 1mM N-acetylcysteine (Sigma-Aldrich), 50 ng/mL EGF (PeproTech, Cranbury, NJ, USA), 100 ng/mL Noggin (PeproTech), 500 ng/mL R-spondin-1 (PeproTech), 100 ng/mL Wnt 3a (PeproTech), 10 nM gastrin (Sigma-Aldrich), 10 mM nicotinamide (Sigma-Aldrich), 500 nM A83-01 (TGFβ type I receptor inhibitor; Tocris), 10 μM SB202190 (P38 inhibitor; Sigma-Aldrich), and 8% fetal bovine serum (Atlanta Biologicals, Lawrenceville, GA, USA), supplemented with 10 μM Rho-associated kinase inhibitor (ROCKi) Y-27632 (StemGent, Lexington, MA, USA) and 2.5 μM glycogen synthase kinase 3β (GSK3β) inhibitor CHIR99021 (StemGent), was used as the culture medium. The isolated ISCs were grown in Matrigel™ in CMGF+ media to progress differentiation [35]. Though the CMGF+ medium with the ROCKi and GSK3 inhibitors was used for the first two days of ISC culture to boost ISC survival and prevent dissociation-induced apoptosis (anoikis), the CMGF+ medium without the ROCKi and GSK3 inhibitors was utilized after the first two days of culture to drive differentiation of the canine colonoids [35]. Organoids were fully differentiated (Figure 3A) after 6–8 days in differentiation media (CMGF+ without ROCKi and GSK3 inhibitors), exhibiting a luminal compartment, crypt epithelium, and villi-like structures, as well as the exfoliation of denuded epithelia into the lumen [35]. The CMGF+ culture medium was changed every other day, and the organoids were passaged once a week when they were potentially “mature”.

### 2.5. Preparation of 2D Canine Colonic Monolayer and Maintenance

After seven days of culture (Figure 3A), the 3D colonoids were harvested from the Matrigel^®^ using the Corning™ cell recovery solution. Matrigel^®^ plugs were dissociated by repeated pipetting after adding 500 μL of pre-chilled cell recovery solution into each well, and the plate was incubated for 30 min at 4 °C to dissolve the Matrigel^®^. The mixture was then transferred to a 15 mL tube and centrifuged at 100× *g* for 5 min at 4 °C. The organoid pellet was resuspended in 1 mL TrypLE Express and incubated at 37 °C for 10 min with shaking. To stop the TrypLE action, 6 mL of DMEM/F12 was added to the mixture before centrifugation at 100× *g* for 5 min at 4 °C. The supernatant was removed, and the pellet was resuspended with CMGF+ media. The harvested colonoid cell suspension was filtered through a sterile cell strainer (40 µm nylon mesh, Thermo Fisher Scientific) to obtain a single-cell suspension. The viability of dissociated cells was assessed by the trypan blue exclusion test [58,59], and cells were counted manually using a hemocytometer (Hausser Scientific, Horsham, PA, USA). The Transwell™ inserts were pre-coated with Matrigel^®^ (100 μg/mL; Corning^®^) and rat tail collagen I (30 μg/mL; Fisher Scientific) to promote adhesion, growth, and differentiation in CMGF+ media at 37 °C for 1 h. Dissociated colonoid cells (Passages #15–20) were seeded at a density of 100,000 cells/cm^2^ in pre-coated inserts and allowed to grow and differentiate in the presence of CMGF+ media for 10 to 12 days before the experiments (Figure 3B). The CMGF+ culture medium was changed every other day. The structural integrity of the cell monolayer was monitored every other day for up to two weeks by phase-contrast microscopy. Colonoid-derived monolayers and colonoids were fixed and processed for histology and/or TEM, as described in prior studies [35,36,60].

### 2.6. RNA Extraction and Quantitative Real-Time Polymerase Chain Reaction Analyses

After adding TRIzol RNA isolation reagent (ThermoFisher Scientific) to each monolayer (when they attain a steady transepithelial electrical resistance (TEER) value, i.e., Day 11 for colonoid-derived monolayers and Day 19 for Caco-2-derived monolayers) and pipetting up and down, the cellular contents were removed for total RNA extraction using the TRIzol method as described by the manufacturer. After extraction, total RNA samples were purified using the RNeasy MinElute Cleanup Kit (QIAGEN, Hilden, Germany) following the manufacturer’s instructions. Purified RNA samples were quantified and assessed for quality using a Bioanalyzer 2100 instrument (Agilent Technologies, Palo Alto, CA, USA). A total of 0.5 µg of purified RNA was used to synthesize first-strand cDNA according to the manufacturer’s instructions using the SuperScript III first-strand synthesis kit (Invitrogen, Waltham, MA, USA).

qPCR for the expression of intestinal epithelial cell markers, tight and adherens junction proteins, transporters, and cytochrome P450 (CYP) enzymes (primers listed in Table 1) was carried out using the synthesized complementary DNA (cDNA), which contains only the coding DNA sequences, using PowerUp SYBR Green Master Mix following the manufacturer’s protocol using QuantStudio™ 3 (Applied Biosystems by Thermo Fisher). The thermocycling conditions were as follows: 50 °C for 2 min and then 95 °C for 2 min, followed by 35 cycles of 95 °C for 15 s, 55 °C for 15 s, and 72 °C for 1 min. The expression of each gene of interest was normalized using glyceraldehyde-3-phosphate dehydrogenase (GAPDH) and quantified using the delta–delta Ct method [38]. The gene expression results are displayed as normalized Ct (dCt) to GAPDH. A canine liver sample was used for RNA extraction and cDNA synthesis for use as a positive control for the Oatp2b1 expression studies.

### 2.7. Assessment of Monolayer Integrity by TEER Measurement and Fluorescein Isothiocyanate-Dextran Leakage Assay

TEER is a quantitative technique that measures the electrical resistance of the monolayers, reflecting the ionic conductance of the paracellular pathway in each monolayer [61], and is indicative of the integrity of the cell monolayer. A Millipore Millicell^®^ ERS-2 epithelial volt–ohm meter was used to measure the TEER values daily. The TEER measurements of individual monolayers (R_sample_) were normalized by comparison to a cell-free insert (R_blank_) and multiplied by the area of the insert (0.33 cm^2^) as follows (Equation (1)):

TEER (Ω×cm^2^) = [R_sample_ − R_blank_] × 0.33 cm^2^ (1)

TEER (Ω×cm^2^) measurements were performed in all experimental replicates for both Caco-2 cell and canine colonoid monolayers. Prior to incubation with the model drugs, only monolayers with steady-state TEER values [36] above 500 Ω×cm^2^ and 4000 Ω×cm^2^ were utilized for Caco-2 cells and colonoid-derived monolayers, respectively [9,62,63].

In addition to the TEER values, the canine colonoid monolayer integrity was evaluated with the commonly used zero permeability compound 4 kDa FITC-dextran [61]. Since FITC-dextran does not impact the permeability of metoprolol or the integrity of the monolayer, FITC-dextran was combined with metoprolol to simultaneously confirm the TJ structural integrity of the canine colonoid monolayer [64]. FITC-dextran concentrations over time were measured using a SpectraMax^®^ M2e (Molecular Devices, San Jose, CA, USA) microplate reader at excitation and emission wavelengths of 490 and 520 nm, respectively [13]. Briefly, the donor (AP) side (pH 6.8) contained 200 µg/mL FITC-dextran with or without metoprolol (0.4 mg/mL). The receiver (BL) side contained the HBSS transport buffer at a pH of 7.4. The percentage of relative fluorescence units (%RFU) was measured for the donor and receiver sides of the wells at 120 min, as previously described [13].

### 2.8. Preparation of Transport Buffer and Drug Solutions

Stock solutions of the test drugs (metoprolol, atenolol, and propranolol) were prepared in distilled water. Working solutions were prepared in the appropriate transport buffer for use at the pH (6.8 or 7.4) of the in vivo condition being replicated. All assays were performed in triplicates. Hank’s balanced salt solution (HBSS) buffer with Ca^2+^ and Mg^2+^ was used as a transport buffer (GIBCO™). The inclusion of Ca^2+^ and Mg^2+^ in the buffer prevents monolayer detachment from the filter. The pH of the transport buffer was adjusted to pH 6.8 or 7.4 with 1N HCl or 1N NaOH before use. Final drug concentrations were 0.04 mg/mL and 0.4 mg/mL metoprolol, 0.2 and 2.0 mg/mL atenolol, and 1.0 mg/mL propranolol. The physicochemical characteristics of the three tested drugs are presented in Table 2, based on the information available in PubChem [65].

### 2.9. Bidirectional Transport Experiments

All transport experiments were conducted at room temperature following the conditions outlined in earlier reports during sampling and incubation [66,67,68]. The culture media were aspirated from both AP (0.2 mL) and BL (0.7 mL) chambers. For both the canine colonoid and the Caco-2 monolayer, the BL chambers with HBSS buffer at pH 7.4 (0.7 mL) and the AP chambers with appropriate HBSS buffer at pH 6.8 or 7.4 (0.2 mL) (canine) or pH 6.8 (0.2 mL) (Caco-2 cells) were rinsed and equilibrated at room temperature for 30 min. Two to three wells per drug/pH/direction were used for data generation.

#### 2.9.1. Apical-to-Basolateral (AP→BL) Permeability

After Caco-2 and canine colonoid monolayers were equilibrated in the transport buffer for 30 min, the buffer was aspirated from the AP (0.2 mL) and BL (0.7 mL) chambers. The media of the AP chamber were replaced with 0.2 mL of the drug solution prepared in an appropriate transport buffer. HBSS buffer with pH 7.4 was added to the BL chambers. At pre-defined time points (15, 30, 45, 60, 90, and 120 min), samples of 100 µL buffer were collected from the receiver chamber (BL) and replaced with the same volume (i.e., 100 µL) of transport buffer at pH 7.4. For the AP side, drug concentrations of the donor solution were measured prior to introduction into the well and at the end of the study (120 min). In so doing, no buffer replacement was needed. All study samples were labeled and stored at −80 °C until further analysis.

#### 2.9.2. Basolateral-to-Apical (BL→AP) Permeability

The same procedures described above for studying AP*→*BL permeability were followed when studying BL*→*AP, with the only difference being that the donor chamber was now the BL side and the receiver chamber was now the AP side. At pre-determined time intervals (15, 30, 45, 60, 90, and 120 min), 100 µL of buffer was removed from the receiver chamber (AP) and replaced with the same volume (i.e., 100 µL) of transport buffer with the specified pH. The collected samples were stored at −80 °C until further analysis by mass spectrometry (details below).

### 2.10. Sample Preparation for Mass Spectrometer Analysis

All the experimental samples in HBSS buffer were prepared by transferring 40 µL of buffer into a 1.5 mL polypropylene tube. A volume of 160 µL of acetonitrile was added to each sample, and all samples were vortexed for 30 s. Samples were diluted in water based on the expected concentration in the donor or receiver wells. Samples containing atenolol were diluted into a linear range of 0.5–30 µg/mL. Specimens containing metoprolol were diluted into a linear range of 0.2–40 µg/mL, and samples containing propranolol were diluted into a linear range of 1–300 µg/mL. A calibration curve and three quality control (QC) sample replicates were prepared in pH 6.8 HBSS buffer and pH 7.4 HBSS buffer for each analyte. Calibration curves and QCs were prepared and diluted the same way as the samples. All calibration curves had an R^2^ value of 0.99, and all QC samples showed a bias of less than 10% (Supplemental Figure S1 and Table 3). The area ratio in the calibration curve was calculated by the instrument data processing software (Thermo Xcalibur version 4.1.50, Thermo Fisher Scientific Inc., Waltham, MA, USA). It is defined as the peak area of the compound divided by the peak area of the labeled internal standard.

#### 2.10.1. Preparation of Standards and Solutions

Metoprolol acid-d_5_, atenolol, atenolol-d_7_, and propranolol HCl analytical standards were purchased from Toronto Research Chemicals (Toronto, ON, CA). Metoprolol was purchased from Sigma-Aldrich (St. Louis, MO, USA). Propranolol-d_7_ was purchased from Cerilliant^®^ (Round Rock, TX, USA) as a 100 ng/µL solution in methanol. All stock solutions were prepared at 1 mg/mL in methanol or 50/50 (*v*/*v*) methanol/water, except for propranolol-d_7_, which was purchased in solution at a concentration of 100 ng/µL.

#### 2.10.2. Analytical Method

A Vanquish™ Flex LC pump interfaced with a TSQ Altis mass spectrometer (MS) (Thermo Scientific™, San Jose, CA, USA) was used to analyze all analytes. The column used for all analyses was a Hypersil GOLD™ aQ Vanquish 50 × 2.1, 1.9 µm (Thermo Scientific™). Mobile Phase A was water plus 0.1% formic acid, and Mobile Phase B was acetonitrile plus 0.1% formic acid for all analyses. The column compartment temperature was 40 °C for all analyses. The chromatographic gradient was as follows: start 0% organic, linear ramp from 0.5 to 2 min up to 80% organic (100% organic for propranolol), hold at 80% organic (100% organic for propranolol) for 0.4 min, return to starting conditions for 0.01 min, and hold at starting conditions for 0.59 min. The solvent flow rate was 0.4 mL/min. A 2 µL injection volume was used.

The following MS method parameters were used for the analysis of all analytes. The resolution of Q1 and Q3 was 0.7 FWHM. The CID gas was set to 1.5 mTorr. The chromatographic peak width was 2 s, and the cycle time was 0.2 s. The source conditions were as follows: positive mode spray voltage 2000 V, sheath gas 60 Arb, auxiliary gas 22 Arb, sweep gas 1 Arb, ion transfer tube temperature 350 °C, and vaporizer temperature 350 °C. The total run time of the method was 3 min.

### 2.11. P_app_ Calculations

The uncorrected *P_app_* estimates for the three drugs of interest (*P_app-uncorrected_*, cm/s) were calculated as follows (Equation (2)) [7]:

(2) $$P_{app-uncorrected}\;=\frac{V_{R}}{\left(A\times C_{0}\right)}\times \frac{dC}{dt}$$

where *V_R_* is the volume in the receiver chamber (mL), A is the surface area of the filter ($0.33\;{\mathrm{cm}}^{2}$), *C*_0_ is the initial drug concentration (µM) in the donor chamber, and $\frac{dC}{dt}$ is the maximum slope of the line defining the cumulative drug concentration (mg/mL) vs. time (min) in the receiver compartment. Drug concentrations in the donor and receiver chambers at designated time points were measured by mass spectrometry (see the details above in Section 2.10.2). The estimated concentrations in the receiver compartment were corrected for drug removed during prior samples. The efflux ratio was calculated as *P_app_*_,BL-AP_/*P_app_*_,AP-BL_, where a ratio greater than 2 indicates active efflux [69,70].

These *P_app_* value estimates were further corrected for the percent recovery (*Rec* (%)) in each well [71,72] as follows (Equation (3)):

(3) $$Rec\;(\%)\;=\frac{mg\;donor\;hr\;2+total\;mg\;receiver\;hr\;2}{mg\;drug\;introduced\;into\;the\;donor\;compartment\;at\;hr\;0}$$

The corrected *P_app_* estimates reported herein were, therefore, calculated as follows (Equation (4)):

(4) $$P_{\mathrm{app}}=\;\frac{Papp-uncorrected\;}{Rec\;\left(\%\right)}$$

The slope of the relationship between the sampling time and the corrected *P_app_* values were calculated as the least-squares estimate of the linear regression line defining the drug transfer rate from the first quantifiable drug concentration in the donor cell to 120 min.

### 2.12. Statistics

Since only two wells were tested per monolayer type and assay condition in this exploratory study, there was insufficient power to assess the statistical significance of the *P_app_* differences between the model systems. Therefore, descriptive statistics (arithmetic mean, SD, SEM, CV%) were used to summarize *P_app_* estimates for the various experimental conditions in this preliminary study. The comparative expression of transporters and CYP enzymes’ expression in Caco-2-derived and canine-colonoid-derived monolayers was performed with GraphPad Prism 9 (Version 9.4.1) (https://graphpad.com/; accessed on 11 January 2023) using a one-way ANOVA with Šídák’s adjustment for multiple-comparisons or a two-tailed Student’s *t*-test, as *p*-values <0.05 were considered statistically significant for all analyses.

## 3. Results

### 3.1. Assessment of Monolayer Integrity

**Figure 4:**
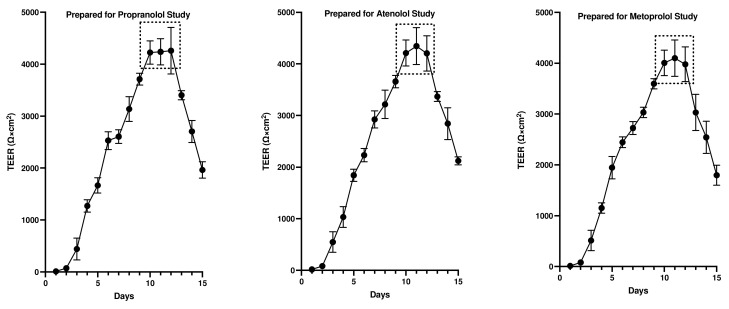
Evaluation of canine-colonoid-derived monolayer integrity. Continuous TEER (Ω×cm^2^) analysis over 15 days was used to assess the integrity of each colonoid monolayer. Monolayers were prepared for propranolol, atenolol, and metoprolol studies. Values are expressed as the arithmetic mean of the data and one standard deviation of N = 18 monolayers.

**Figure 5:**
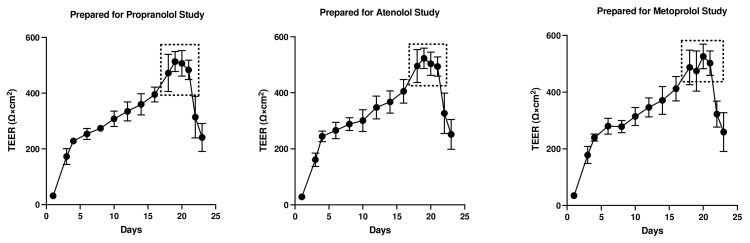
Evaluation of Caco-2 cell monolayer integrity. Continuous TEER (Ω×cm^2^) analysis of the monolayer over 23 days was used to assess the permeability of propranolol, atenolol, and metoprolol. Values are expressed as the arithmetic mean of the data and one standard deviation of N = 12 monolayers.

The TEER (Ω×cm^2^) values were recorded for both colonoids and Caco-2 cells to determine the integrity and confluence of the monolayer, as reported in Figure 4 and Figure 5. Before initiating the permeability studies, the wells containing the 2D canine colonic monolayer were maintained for 11 days. The colonic monolayer TEER values began to plateau on Days 10–12 at an average of 4198.3 ± 142.4 Ω×cm^2^ (mean ± SD) (Supplemental Table S1A). Caco-2 cell monolayers were monitored for a total of 23 days. The TEER values began to plateau around Days 18–21, with an average of 501.4 ± 15.6 Ω×cm^2^ (mean ± SD) (Supplemental Table S1B).

In the colonoid monolayer, FITC-dextran was combined with metoprolol to assess monolayer integrity and measure the permeability of metoprolol simultaneously. For FITC-dextran without metoprolol, the percentage of relative fluorescence was estimated at 93.7% RFU and <0.02% RFU in the donor and receiver chambers, respectively. In comparison, these estimates were 94.6% relative fluorescence (RFU) (donor side) and <0.02% RFU (receiver side) for FITC-dextran with metoprolol (Table 4). This suggests that the FITC-dextran transport was less than 0.02% both with and without metoprolol, thus confirming the integrity of the colonoid monolayer [38].

### 3.2. Drug-Specific P_app_ Estimates

The estimated *P_app_* values per drug, direction, pH, and system are provided in Table 5 (see Supplemental Table S2 for the estimated drug concentration).

Propranolol (1 mg/mL)

**Figure 6:**
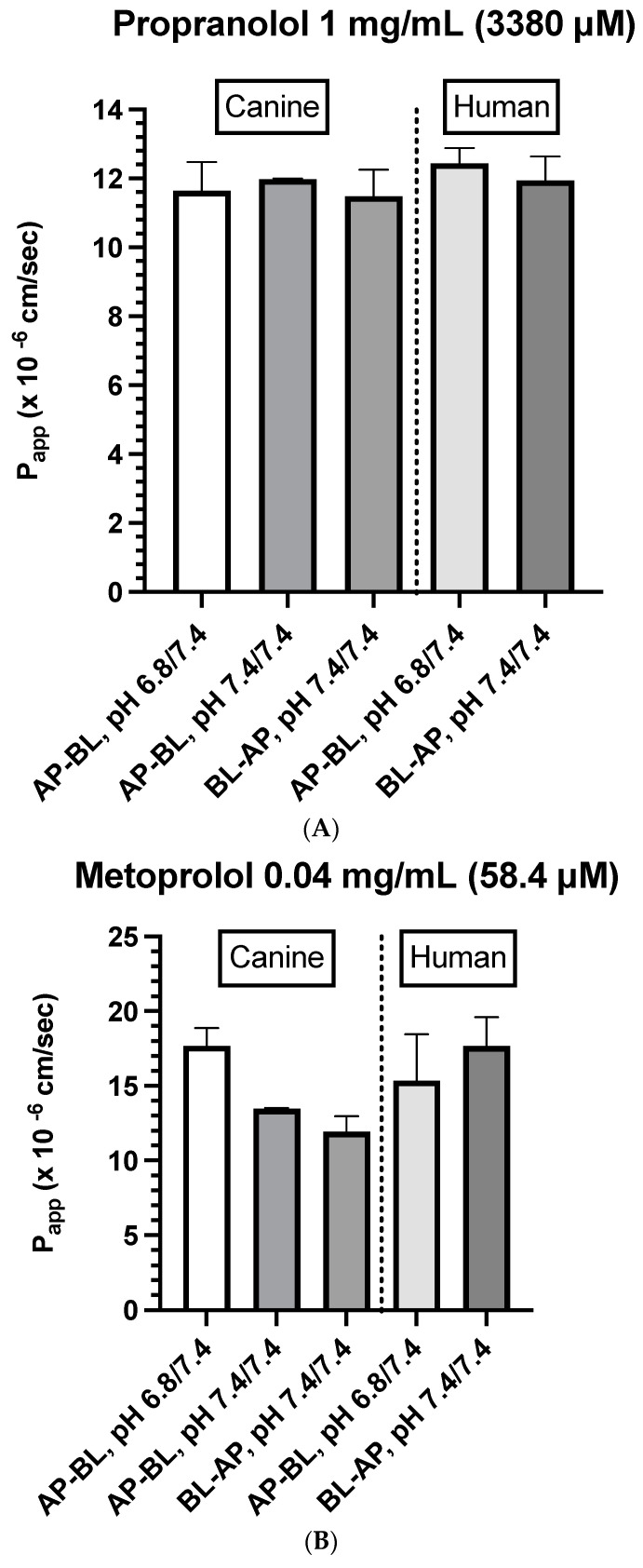

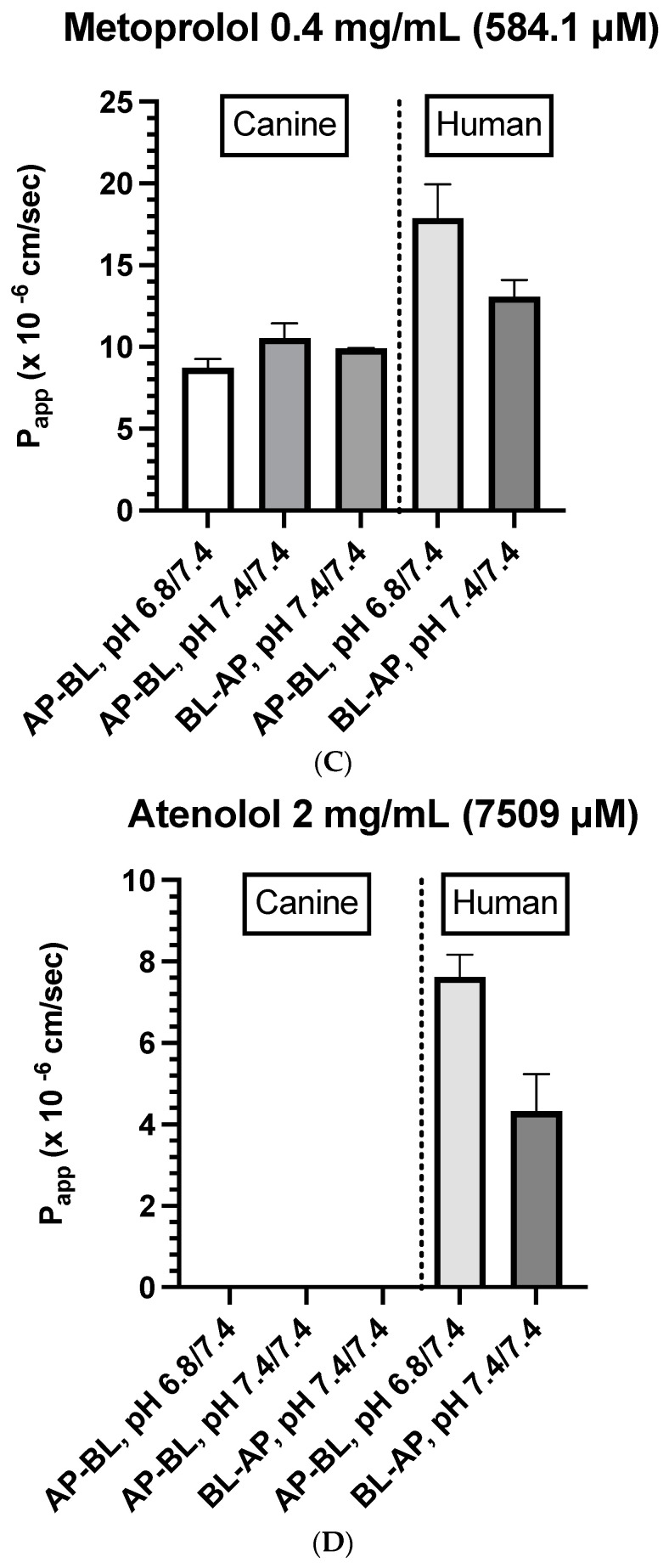
(**A**) Comparison of propranolol *P_app_* estimates in canine colonoid vs. human Caco-2 monolayers as a function of transport direction (AP-BL vs. BL-AP) and apical pH (6.8 vs. 7.4). (**B**) Comparison of metoprolol (0.04 mg/mL) *P_app_* estimates in canine colonoid vs. human Caco-2 monolayers as a function of transport direction (AP-BL vs. BL-AP) and apical pH (6.8 vs. 7.4). (**C**) Comparison of metoprolol (0.4 mg/mL) *P_app_* estimates in canine colonoid vs. human Caco-2 monolayers as a function of transport direction (AP-BL vs. BL-AP) and apical pH (6.8 vs. 7.4). (**D**) Comparison of atenolol (2 mg/mL) *P_app_* estimates in canine colonoid vs. human Caco-2 monolayers as a function of transport direction (AP-BL vs. BL-AP) and apical pH (6.8 vs. 7.4).

- Canine colonoids: The bidirectional *P_app_* values were similar irrespective of the pH of the apical (donor) side (Table 5 and Figure 6A). To confirm that pH does not impact the results, data were collected at two donor pH values (6.8 and 7.4). No pH-associated differences were observed.
- Caco-2: To confirm that pH does not impact the movement of propranolol, the transcellular AP→BL movement was evaluated in the human Caco-2 cell line. Estimated *P_app_* values for the Caco-2 monolayers and dog colonoids were similar (Table 5 and Figure 6A).

Metoprolol (0.04 and 0.4 mg/mL)

- Canine colonoids: The AP→BL *P_app_* values were lower in the medium containing 0.4 vs. 0.04 mg/mL metoprolol. Moreover, at the 0.04 mg/mL concentration, the movement from AP→BL was slightly greater than that from BL→AP. The transport from AP→BL and BL→AP tended to be similar when evaluated at the 0.4 mg/mL concentration. Upon considering the data generated across the two metoprolol concentrations, the pH of the apical chamber did not consistently influence the magnitude of the *P_app_* estimate (Figure 6B,C and Table 5).
- Caco-2: Unlike canine colonoids, the *P_app_* values were not markedly influenced by metoprolol concentration, and at both concentrations, the movements from AP→BL and BL→AP were comparable (Figure 6B,C and Table 5). The concentration-associated differences in the *P_app_* values seen with the colonoid were not observed with the Caco-2 monolayer. Moreover, although the two cell line monolayers exhibited similar AP→BL *P_app_* values in the presence of 0.04 mg/mL metoprolol, the AP→BL *P_app_* values for the Caco-2 monolayer tended to be higher than that of the canine colonoid when the donor concentration was increased to 0.4 mg/mL. Although the movement from AP→BL was somewhat greater than that seen in the BL→AP direction in the colonoid (0.04 mg/mL metoprolol, but not at the 0.4 mg/mL donor concentration), that difference was not seen with the 0.04 mg/mL concentration or was only minimally appreciated at the 0.4 mg/mL concentration when the Caco-2 monolayer was used. When considering the variability across observations and the small number of wells tested, statistical inferences should not be linked to these outcomes (Figure 6C,D and Table 5). Thus, unlike propranolol, differences in the behavior of metoprolol were seen when comparing the two cell line systems. However, these preliminary findings should be interpreted cautiously in light of our limited sample size and the background variability in our system.

Atenolol (0.2 mg/mL)

For atenolol (0.2 mg/mL), the transport was not quantifiable in either direction for the canine colonoid and the Caco-2 monolayer (Table 5). However, because the values were below the analytical limit of quantification, we increased the donor concentration 10-fold (i.e., 2.0 mg/mL) to ensure that we were able to determine if some paracellular movement did, in fact, occur across either of the two monolayers:

Atenolol (2.0 mg/mL)

- Canine colonoids: Atenolol was not detectable in the receiver compartment, irrespective of the experimental pH or the direction of drug transport, i.e., AP→BL or BL→AP (Figure 6D and Table 5).
- Caco-2: Incubation of Caco-2 cells with 2.0 mg/mL atenolol resulted in measurable concentrations in the receiver compartment for both AP→BL and BL→AP directions, with higher *P_app_* estimates, reported after incubation in the apical chamber (Figure 6D and Table 5). These preliminary findings suggest a somewhat greater ability for atenolol to undergo paracellular transport across the Caco-2 monolayer as compared to that of the dog colonoid. However, at the lower atenolol concentration, neither system was associated with quantifiable movement from the donor to receiver compartment (irrespective of direction).

### 3.3. Gene Expression Analyses

**Figure 7:**
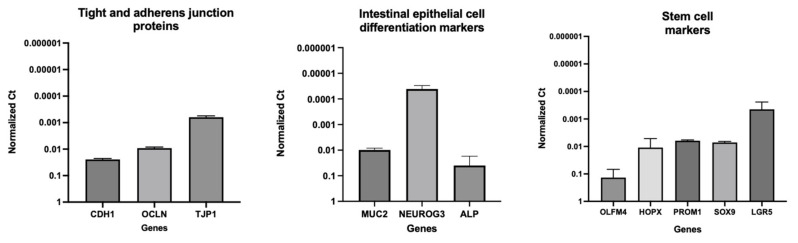
Expression of tight and *adherens* junction proteins, intestinal epithelial cell differentiation, and stem cell markers in canine-colonoid-derived monolayers. The gene expression results are displayed as normalized Ct (dCt) to glyceraldehyde-3-phosphate dehydrogenase (GAPDH) (see Table 6 for Ct values). Values are expressed as the arithmetic mean of the data and one standard deviation of N = 3 monolayers. Cadherin 1 (CDH1); occludin (OCLN); tight junction protein 1 (TJP1); mucin 2 (MUC2); neurogenin 3 (NEUROG3); intestinal alkaline phosphatase (ALP); olfactomedin 4 (OLFM4); HOP homeobox (HOPX); prominin 1 (PROM1); SRY-box transcription factor 9 (SOX9); leucine-rich repeat-containing G-protein-coupled receptor 5 (LGR5).

**Figure 8:**
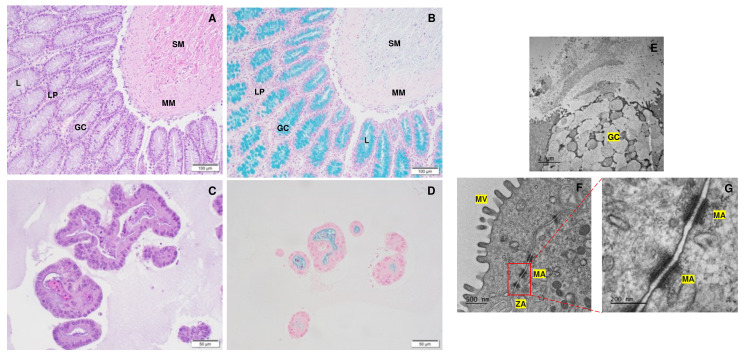
Visualization of goblet cells containing mucus in canine colon tissue and colonoids. (**A**) Histological images of hematoxylin and eosin (H&E) staining of colon tissue. (**B**) Alcian blue staining of colon tissue revealed goblet cells containing mucus. (**C**) The H&E staining of colonoids at five days after passage. (**D**) Colonoids at 5 days after passage with mucus production are shown by Alcian blue staining. (**E**) A representative transmission electron microscopy (TEM) image of colonoid shows a goblet cell with mucin granules. (**F**) A TEM image of the intercellular junctional complex in canine colonoid. The microvilli of the canine colonoid are visible at higher magnifications using TEM than with a standard optical light microscope [74]. (**G**) A zoom-in that shows a high-power magnification of the red dashed area in “F”. Submucosa (SM); Muscularis mucosa (MM); Lamina propria (LP); rows of goblet cells (GC) are oriented toward the gland lumen (L); mucus-filled goblet cell (GC); microvilli (MV); zonula adherens (ZA); desmosome or macula adherens (MA).

**Figure 9:**
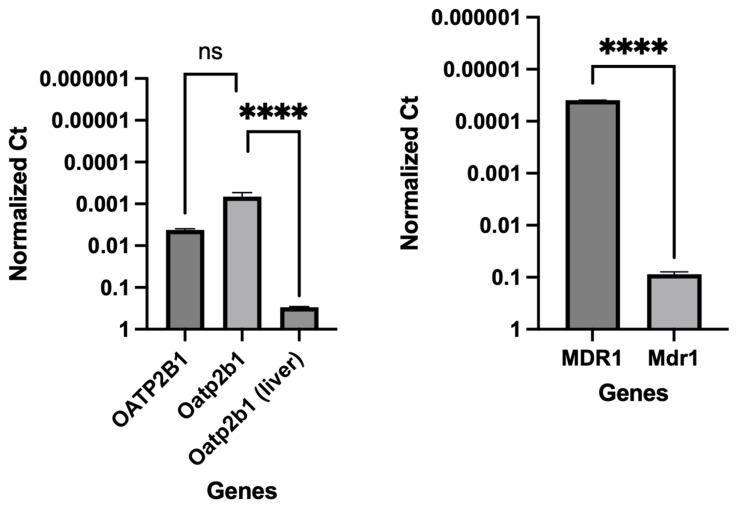
Comparative expression of transporters in Caco-2-derived and canine-colonoid-derived monolayers. Organic anion transporting polypeptide (OATP2B1, human; Oatp2b1, dog); multidrug resistance p-glycoprotein (MDR1, human; Mdr1, dog). A canine liver sample was used for RNA extraction and cDNA synthesis for use as a positive control for Oatp2b1 expression studies. Gene expression results are displayed as normalized Ct (dCt) to glyceraldehyde-3-phosphate dehydrogenase (GAPDH) (see Table 6 for Ct values). Values are expressed as the arithmetic mean of the data and one standard deviation of N = 3 monolayers. Between-groups statistics were performed with GraphPad Prism 9 (Version 9.4.1) (https://graphpad.com/; accessed on 11 January 2023) using one-way ANOVA with Šídák’s adjustment for multiple-comparisons (for OATP2B1) or a two-tailed Student’s *t*-test (for MDR1). *p*-values < 0.05 were considered statistically significant for all analyses. **** *p* < 0.0001. ns = no significance difference.

**Figure 10:**
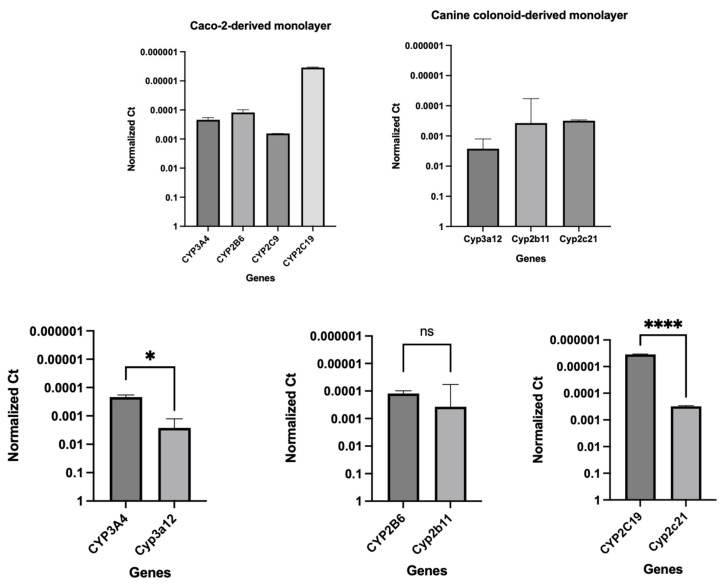
Comparative expression of CYP enzymes in Caco-2-derived and their orthologs in canine-colonoid-derived monolayers. Gene expression results are displayed as normalized Ct (dCt) to glyceraldehyde-3-phosphate dehydrogenase (GAPDH) (see Table 6 for Ct values). Values are expressed as the arithmetic mean of the data and one standard deviation of N = 3 monolayers. CYP enzymes in human Caco-2 (CYP3A4, CYP2B6, CYP2C9, and CYP2C19); CYP enzymes in canine colonoids (Cyp3a12, Cyp2b11, and Cyp2c21). Group comparisons were performed with GraphPad Prism 9 using a two-tailed Student’s *t*-test. *p*-values < 0.05 (*) were considered statistically significant for all analyses. **** *p* < 0.0001. ns = no significance difference.

The integrity and differentiation of the colonoid-derived monolayers were confirmed by measuring the expression of tight and *adherens* junction proteins, including CDH1, OCLN, and TJP1, as well as intestinal epithelial cell differentiation markers such as MUC2 (for goblet cells), NEUROG3 (for enteroendocrine cells), and ALP (for absorptive epithelium) (Figure 7; Table 6). Additionally, stem cell markers such as OLFM4, HOPX, PROM1, and SOX9 were detected in colonoid-derived monolayers (Figure 7; Table 6; see the caption of Figure 7 for the definition of the abbreviations). The electron microscopic images of colonoids also clearly showed the presence of goblet cells containing mucin granules (Figure 8). The expression of key intestinal epithelial transporter molecules, such as Mdr1 and Oatp2b1, and important CYP enzymes expressed in the intestine, such as Cyp3a12, Cyp2b11, and Cyp2c21, was measured in the canine-colonoid-derived monolayers and compared to the expression of their orthologs in human Caco-2-derived monolayers. In canine-colonoid-derived monolayers, the expression of Mdr1, Cyp3a12, and Cyp2c21 was increased relative to their orthologs in Caco-2-derived monolayers, as expected (Figure 9 and Figure 10; Table 6). We also used qPCR to evaluate transporter and CYP metabolic enzyme expression in other canine cell lines utilized for drug uptake study, such as MDCK cells, which demonstrated low expression of these markers. Our findings were consistent with those of prior research [73]; hence, this was not included in the study.

## 4. Discussion

Identifying the permeability characteristics of an orally administered drug is one of the critical steps in predicting GI drug absorption. This information helps identify factors that can influence oral bioavailability and guide formulators in their efforts to optimize the fraction of the administered dose that is absorbed. While in vitro tools for exploring drug permeability and enterocyte drug metabolism are available for human therapeutics, there is no corresponding tool available that faithfully models the canine GI tract. In other words, to date, there are no systems available to interrogate canine intestinal passive permeability, transporter activity, or enterocyte metabolism. A completely differentiated and confluent Caco-2 monolayer requires approximately three weeks of culture, whereas the present study demonstrated that intestinal organoid-derived monolayers require significantly less time. Moreover, as we showed in our study, it is inappropriate to simply extrapolate Caco-2 data to the dog. Rather, there is a need for a canine-specific tool. Such a tool is provided by the canine 3D organoids. By developing this system, we can further explore how absorption through the intestinal membrane varies as a function of GI segment. Accordingly, the current study provided a first step in the development of a novel in vitro system that can be used to address a range of questions relevant to canine medicine and to explore potential bias that may occur when relying upon predictions based on interspecies extrapolations (dog to human or human to dog). We studied the feasibility of using canine-colonoid-derived 2D monolayers for an apparent permeability assessment, focusing first on the passive diffusion of drugs across the canine enterocyte membrane.

Generally, we expect that passive transcellular permeability characteristics are translatable across biological membranes. This assumption is supported by the use of the MDCK cells and Lewis lung carcinoma-porcine kidney cells (LLC-PK_1_) for the evaluation of passive absorption mechanisms [75]. However, differences between cell lines may exist when assessing paracellular absorption [55,76]. Therefore, we explored the feasibility of the canine colonoid system using three well-characterized β-blockers with transcellular or paracellular absorption and determined the validity of our assumptions by comparing canine colonoid *P_app_* values to those obtained when the same compounds were tested using the Caco-2 cell system. If differences in *P_app_* estimates between systems were identified, then the use of Caco-2 data to predict canine drug intestinal passive permeability would need to be further examined.

Consistent with our expectations, we observed comparable absorption (AP→BL) and secretion (BL→AP) characteristics across the two systems for propranolol. In contrast, system dissimilarities were observed for metoprolol. For example, while the concentration of metoprolol influenced colonoid *P_app_* values across both AP→BL and BL→AP directions, such differences were not seen when using the Caco-2 monolayer. Furthermore, *P_app_* values in both directions were higher for the Caco-2 monolayer when the donor concentration was 0.4 mg/mL as compared to that of the colonoid. In contrast, when the donor concentration was 0.04 mg/mL, AP→BL (apical pH = 6.8), the *P_app_* values were comparable. The only movement in the BL→AP direction was greater for the Caco-2 vs. canine colonoid monolayer systems.

Although we assumed that transcellular permeability would be similar across species and tissues, one possible reason for the observed differences between Caco-2 and colonoids could be unanticipated regional differences across the enterocytes lining the various intestinal regions and differential expression of transporters and CYP enzymes. This possibility was explored by Incecayir et al. [77] and Zur et al. [78]. Incecayir et al. estimated metoprolol *P_eff_* values using an in situ single-pass intestinal perfusion system (SPIPS) in mice and/or rats [77]. They observed that the metoprolol intestinal permeability of both species was higher in the distal ileum vs. the jejunum. Recognizing that *P_eff_* values factor surface area into its estimate, this outcome likely reflects segmental surface area differences or dissimilarity in intestinal mucus boundary layers. However, in contrast to the aforementioned rodent study, using a SPIPS study design in human subjects, Dahlgren et al. detected no statistically significant regional differences in metoprolol *P_eff_* in the colon vs. the ileum [79]. Altogether, these results are inconsistent with a possible influence of the cellular configuration of the Caco-2 cell vs. canine colonoid.

With regard to potential transporter involvement, Incecayir et al. confirmed that metoprolol is not a substrate for P-gp transport, excluding the notion that observations were due to differences in efflux transporter expression [77]. Moreover, if P-gp transporters were in fact involved, it would have negatively influenced movement from AP→BL (decrease in *P_app_* values), but positively increased *P_app_* values in the BL→AP direction. This is not consistent with what was observed experimentally, either at a donor concentration of 0.04 mg/mL or for the BL→AP *P_app_* values (colonoid vs. Caco-2 results) at the donor metoprolol concentration of 0.4 mg/mL. Factoring this point along with our correction for drug loss during our study, we can reasonably assume that the expression of efflux transporters (or losses associated with enterocyte drug metabolism) cannot explain the differences seen between the two systems.

Another point considered in the SPIPS studies by Incecayir et al. [77] and Zur et al. [78] was the potential influence of pH on metoprolol permeability. Although a higher pH tended to be associated with higher *P_eff_* values, this difference was not statistically significant in the rat (N = 6). In contrast, in the rat SPIPS study conducted by Zur et al., markedly higher *P_eff_* values were observed as the perfusate pH was raised from 6.5 to 7.5 (N = 6 per experiment) [78]. In addition, both Incecayir et al. [77] and Zur et al. [78] reported a significant pH-associated change in permeability using either the Caco-2 monolayer or octanol/buffer partition coefficient (and PAMPA membrane) [78]. In all cases, the permeability of metoprolol decreased as the donor pH decreased. The investigators attributed this pH effect to metoprolol being a basic secondary amine that serves as its only ionizable center. As a result, the fraction unionized of metoprolol is negligible at a low pH (i.e., at pH values less than the corresponding pKa) and gradually increases as the pH rises. Incecayir et al. suggested that the small effect of pH on in vivo absorption vs. in vitro permeability may have been attributable to the in vivo presence of a mucous layer, which retains the microclimate pH, regardless of the luminal pH [77]. Interestingly, however, we observed the opposite effect of pH on our 0.04 mg/mL metoprolol *P_app_* values, where there tended to be a decrease rather than an increase as the pH was raised from 6.8 to 7.4. Therefore, again, the reasons for our observations with metoprolol are not readily apparent.

Finally, unlike that of the Caco-2 monolayer or colonoid permeability studies conducted at a donor metoprolol concentration of 0.04 mg/mL, FITC-dextran was included when the canine colonoid donor concentrations were 0.4 mg/mL. While this fluorescent probe has not been associated with changes in drug transcellular permeability, we do not have the data to exclude that possibility in our current investigation. Therefore, an impact of this design difference between permeability study conditions cannot be excluded. The permeability findings on Caco-2 monolayers from Day 14 with low TEER value [54] indicate that the transport of a hydrophilic marker (FITC-dextran) and hydrophilic drug (atenolol) was significantly increased, whereas the transport of metoprolol remained unchanged. The findings indicate that a hydrophilic drug such as atenolol can cross the leaky intercellular connection via the paracellular channel. This assessment is consistent with the observations of Yang et al. (2007), where palmitoylcarnitine, a compound that opens tight junctions, increased the transport of sotalol (a hydrophilic drug) and of FITC-dextran and produced only a small increase in metoprolol transport, but significantly decreased the TEER, the latter indicating a loosening of the tight junction [54].

Unlike the unanswered questions associated with the metoprolol study results, the reasons for the observations with atenolol may be found in the investigation by Dahlgren et al. [79]. Using an SPIP study design in human subjects, atenolol exhibited a >10-fold lower *P_eff_* in the colon as compared to that of the ileum and a >350-fold lower *P_eff_* in the colon as compared to that in the jejunum [79]. This translated to statistically significantly different drug exposure in vivo values (expressed as the area under the concentration time curve (AUC)) when doses were administered to the colon vs. ileum. Extrapolating their observations to our study results and recognizing that the *P_eff_* values were influenced by regional differences in absorptive surface area [80], we cannot exclude the possibility that the differences observed between atenolol *P_app_* in colonoids vs. Caco-2 cells may reflect differences in the TJ expression between these two systems since they represent different intestinal segments showing both enterocyte and colonocyte features [7,8]. Under normal culture conditions, Caco-2 cells can spontaneously undergo morphological and biochemical enterocytic differentiation [7]. The cells become polarized, forming a cell monolayer with apical brush boundary microvilli, tight intercellular junctions, villin expression, and dome formation. When cells approach confluence, the number of proteins characteristic of the colon decreases while the number of proteins characteristic of the enterocytes increases [7]. Consistent with this interpretation is the results of the TEER values we obtained to determine the integrity of the intercellular junctional complex.

The TEER of the monolayers used to assess pre-assay integrity of the monolayers in the present study is comparable to previous reports in Caco-2 cells (Figure 5). In the present study, TEER values greater than 500 Ω×cm^2^ were required for Caco-2 cell monolayers to be considered appropriate for use in transmembrane transport studies [62]. Studies have identified that TEER values in the range of 300–600 Ω×cm^2^ imply the establishment of robust TJs between cells (which are essential for maintaining good monolayer integrity) [9,62,63]. However, the TEER of the Caco-2 cell line is generally higher than that of the in vivo human intestine. Therefore, the passive paracellular pathway in the Caco-2 cells is generally lower than what would be observed in vivo [10].

Based on their relative permeability to small ions, epithelia can be classified as “leaky” or “tight”, as proposed by Machen et al. (1972) [81] and Fromter and Diamond (1972) [82], and this designation is still in use at present [83]. “Leaky” epithelia have higher paracellular small ion permeability than transcellular permeability and low transepithelial resistance, while “tight” epithelia have similar or better transcellular small ion permeability than paracellular small ions with higher transepithelial resistance [81,82]. A “leaky” epithelium [83] has a TEER of less than 100 Ω×cm^2^, indicating greater paracellular permeability, whereas a “tight” epithelium [83] has a TEER of about 2000 Ω×cm^2^, indicating lower paracellular permeability [61,84]. We observed that monolayers obtained from the canine colonoids have eight-times higher TEER values than that of the Caco-2 monolayer, reflecting tighter intercellular junctional complexes than monolayers derived from Caco-2 cells (Figure 4 and Figure 5). That observation is consistent with the higher atenolol *P_app_* values observed in the Caco-2 monolayer as compared with that of the canine colonoids when the 2 mg/mL atenolol concentrations was used. Regarding the difference in *P_app_* estimates obtained with 2 mg/mL vs. 0.2 mg/mL atenolol concentrations, we cannot determine if some movement did in fact occur across the Caco-2 monolayer when testing 0.2 mg/mL atenolol, because all receiver compartment concentrations were below the analytical limit of quantification. Higher P-gp (Mdr1) expression in colonoid-derived monolayers relative to MDR1 in Caco-2 cell monolayers is also a potential explanation for the absence of quantifiable atenolol concentrations in colonoid-derived receiver compartments as atenolol is a substrate for P-gp. This was evidenced in studies showing that co-administration of P-gp inhibitors such as verapamil and zosuquidar decreased the efflux ratio of atenolol [85,86] and that co-administration of another P-gp inhibitor, cyclosporine (a non-specific inhibitor of both efflux and influx transporters) increased the absorption rate of atenolol [85,86].

We also recognize that the *P_app_* values estimated in our study tended to be lower than those reported by others [7,8,10,54,87]. This underscores the importance of comparing permeability results obtained with both systems from the same laboratory. In that regard, variations in permeability results have been observed between laboratories and different Caco-2 culture batches [7,24]. Possible reasons for this observation are the differences in culture and transport protocols between the different research groups [7,42].

Intestinal stem cells (ISCs) can differentiate into progenitor cells, which develop into a diverse range of cell lineages. While a variety of secretory cells (including enteroendocrine cells, goblet cells, and tuft cells) and M cells within Peyer’s patches are all present in the normal canine large intestine [88], tuft cells are only infrequently observed [89]. Therefore, it is important to confirm that the monolayer used in our studies was in fact colonic enterocytes. In the present study, while the expression of mucin 2 (MUC2), intestinal alkaline phosphatase (ALP), and neurogenin 3 (NEUROG3) in the canine-colonoid-derived monolayers validated the differentiation of intestinal epithelial cells (IECs) into different cell subtypes, the expression of olfactomedin 4 (OLFM4), HOP homeobox (HOPX), prominin 1 (PROM1), SRY-box transcription factor 9 (SOX9), and leucine-rich repeat-containing G-protein-coupled receptor 5 (LGR5) genes suggests the presence of intestinal epithelial stem cells [35,38]. PROM1 is a marker for stem cells and early progenitors in the intestine [90]. Both microvilli and brush border enzyme ALP expression also indicate the presence of differentiated enterocytes [35]. OLFM4 is also highly expressed in the crypt base columnar cells in the colon [91]. In our previous research, monolayers generated from canine colonoids were also comprehensively studied for differentiated cell lineages displaying both Ki67 expression and the expression of LGR5, an important marker for adult intestinal stem cells. These monolayers showed NEUROG3 and Chromogranin A (CgA) expression, further confirming the presence of differentiated neuroendocrine cells. Moreover, monolayers contained epithelia covered with mucus-like substances, indicating mucus production [36]. The presence of differentiated goblet cells containing mucus visualized on transmission electron microscopy (TEM) and Alcian blue staining in the present study confirmed the findings from our prior research [36].

Organoid cultures present challenges when investigating microbial interactions due to their unique enclosed architecture surrounded by cells. Different organoid culture systems have been designed to investigate host–microbe interactions, including microinjection into organoids, suspensions outside of organoids, and culture with 2D and 3D organoid monolayers. Organoid-derived monolayer cultures can be established from 3D organoids using a Transwell or flat culture surface in media containing microbes and supplemented with oxygen and various nutrients. Additional studies are needed to optimize culture conditions for both aerobic and anaerobic bacterial populations [92]. Co-culture of bacterial species with the organoids has previously been attempted in mouse and human organoids and has been shown to influence gene expression in the organoids [93]. The microbiome in the canine intestine has been characterized using contemporary molecular techniques and overlaps with the human microbiome taxonomically and functionally to 60–80% of microbiomes in gene content and response to diet [94]. The use of the canine organoids provides an opportunity to examine the effect of gut microbiome on permeability and enterocyte Phase 1 and 2 enzymes. This can be estimated using microbiome characteristics of healthy dogs, as well as that of dogs with inflammatory bowel disease (IBD) to explore the utility of this system in examining gut permeability changes that can affect systemic inflammatory processes. Moreover, as seen in studies of human and murine intestinal organoids, these systems can be invaluable for exploring genetic variations (transporters, intestinal metabolism), drug–drug interactions, and for generating the information needed to support the development of in silico models to predict challenges affecting canine in vivo drug absorption [95]. At this time, we are not considering microbiomes and, therefore, have not incorporated the study on intestinal organoids microbiome interactions. Nonetheless, this is scheduled for the foreseeable future.

The continuation and expansion of this preliminary work are based on the importance of using species-specific monolayers to assess factors such as active influx and efflux transporters, as well as intracellular metabolism on intestinal drug permeability. Moreover, this information should be generated across the various intestinal segments. A first step in supporting the use of such systems is their ability to adequately reflect the passive transport properties (transcellular and paracellular) of the enterocyte membrane.

Despite the limited number of replicates per condition, our study succeeded in providing promising results regarding the utility of canine-derived organoid monolayers for species-relevant assessments of drug passive permeability processes. It also highlighted potential sources of error and the challenges remaining to be addressed. Furthermore, it can influence efforts to extrapolate passive permeability estimates from the Caco-2 cell line to that associated with the canine colon. Clearly, these observed differences warrant future investigations.

## 5. Conclusions

The potential for the usage of canine-colonoid-derived monolayers as a model system for drug permeability assessment was described herein. Despite multiple limitations associated with the use of single-cell monolayers, these systems provide important information on potential barriers to the movement of dissolved drug from the gut lumen to the portal system. Additional studies with organoids derived from other canine intestinal segments and the testing of a broader array of drugs from the BCS classification are needed to conclusively determine the utility of canine-derived intestinal organoid monolayers for predicting the absorption of therapeutic drugs in dogs.

Our preliminary data demonstrated the utility of canine-colonoid-derived monolayers as a model system for the assessment of drug permeability in veterinary medicine. The added value of our method compared with other model systems is that it provides the very first canine-specific intestinal model for evaluating apparent permeability.

## Figures and Tables

**Table 1:** Primers used for real-time PCR expression of tight and adherens junction proteins, intestinal epithelial cell markers, transporters, and cytochrome P450 (CYP) enzymes.

Species	Category	Gene Full Name	Symbol	Forward Primer Sequence (5′–3′)	Reverse Primer Sequence (5′–3′)
*Canis lupus familiaris* (dog)	Housekeeping gene	Glyceraldehyde-3-phosphate dehydrogenase	GAPDH	TCAACGGATTTGGCCGTATTGG	TGAAGGGGTCATTGATGGCG
	Tight and adherens junction proteins	Cadherin 1	CDH1	GACCCAGTAACTAACGACG	CTTCATTCACATCTTCCACG
		Occludin	OCLN	CACTACTGTGTGGTGGATCC	CCTTGTCCCACAATATATTCG
		Tight junction protein 1	TJP1	GAGGGTGATCAAATTCTCAGG	CTGATTCTACAATGCGACG
	Intestinal epithelial cell differentiation markers	Mucin 2	MUC2	CCTGTGCCCCATATTCTGC	GAGATGTTGGAATGGATGCC
		Neurogenin 3	NEUROG3	GAATGCACAACCTCAACTCG	GTAGAGGCTGTGGTCCGC
		Intestinal alkaline phosphatase	ALP	CGTAGTAAACCGCAACTGG	GGAAACATGTACTTTCGGC
	Stem cell markers	Olfactomedin 4	OLFM4	GTATCATGAATGTCAGCAAGC	CTGTAATATTCCAGAATTCTTCC
		HOP homeobox	HOPX	GACCAGGTGGAGATTCTGG	GCCAGACGCTGCTTAAACC
		Prominin 1	PROM1	GATTATTATTTGTGCTGTCC	GAGACTGTAAAGTATTTCCTC
		SRY-box transcription factor 9	SOX9	GTCATCTCCAACATAGAGACC	CTGCTTGGACATCCACACG
		Leucine-rich repeat-containing G-protein-coupled receptor 5	LGR5	GCTAGATCTGTCTTACAACC	GTTCCAGGCTAAATTCAGC
	Transporters	Organic anion transporting polypeptide	Oatp2b1	GATGACTTTGCCCACAACAGC	CAGCAGCAGAGATGAGGAAGC
		Multidrug resistance p-glycoprotein	Mdr1	GTAGCTGAAGAAGTCTTAGCAGC	GCGGCACCAATAGAAATGTTGGC
	Cytochrome P450 (CYP) enzymes	Cytochrome P-450 3a12	Cyp3a12	GATCATGAACATGAAACTTGC	CTTTTCAGGTTGAATAATCCC
		Cytochrome P450 2b11	Cyp2b11	CTGAGGGAGTCCTCCAGGACCC	CACATAGAACAAGTTCATCAGG
		Cytochrome P450 2C21	Cyp2c21 (Cyp2c18)	CAAGCACCTCCTGGATACAGC	CTTCGTGTTCTTTTATTTTTTCC
*Homo sapiens* (human)	Housekeeping gene	Glyceraldehyde-3-phosphate dehydrogenase	GAPDH	TGCACCACCAACTGCTTAGC	GGCATGGACTGTGGTCATGAG
	Transporters	Organic anion transporting polypeptide 2B1/solute carrier organic anion transporter family member 2B1	OATP2B1 (SLCO2B1)	CAAACCTGACTGTGATCCAG	GAGCAGGTTGGCGTATGAGG
		ATP binding cassette subfamily B member 1	ABCB1 (MDR1)	CAGTAGCTGAAGAGGTCTTGGC	CTGTAATAGCTTTCTTTATCCC
	Cytochrome P450 (CYP) enzymes	Cytochrome P450 family 3 subfamily A member 4	CYP3A4	GAGATGGTCCCTATCATTGCC	GATGTTCACTCCAAATGATGTGC
		Cytochrome P450 family 2 subfamily B member 6	CYP2B6	GAAACCGCTGGAAGGTGCTTCG	CTCCTCTATCAGACACTGAGC
		Cytochrome P450 family 2 subfamily C member 9	CYP2C9	GAAGGAGATCCGGCGTTTCTCC	CTTGGTTTTTCTCAACTCCTCC
		Cytochrome P450 family 2 subfamily C member 19	CYP2C19	GATCTGCTCCATTATTTTCC	GTTTTTAAGTAATTTGTTATGG

**Table 2:** Physicochemical characteristics of metoprolol, atenolol, and propranolol.

Drug	Molecular Formula (MF)	Molecular Weight (MW)	Aqueous Solubility (25 °C)	Log P	Dissociation Constants (Basic pKa)
Metoprolol	C_15_H_25_NO_3_	267.36 g/mol	>1000 mg/mL	2.15	9.56
Atenolol	C_14_H_22_N_2_O_3_	266.34 g/mol	13.3 mg/mL	0.16	9.58
Propranolol	C_16_H_21_NO_2_	259.339 g/mol	0.0617 mg/L	3.48	9.53

**Table 3:** Quality control bias (%). Average measured value of quality control samples for all three compounds and bias (%) at the two buffer pH levels; minimum N = 8 for all calculations.

Compound Name	pH	QC Level (ppm)	Average Measured Value (ppm)	Bias (%)
Atenolol	6.8	25	23.1	−7.41
	7.4	25	23.8	−4.60
Metoprolol	6.8	25	25.5	1.93
	7.4	25	24.9	−0.22
Propranolol	6.8	40	40.2	0.61
	7.4	40	40.9	2.33

**Table 4:** Transport of FITC-dextran (expressed in percentage) across the colonoid-derived monolayer. Values are expressed as the arithmetic mean of the data and one standard deviation of 3 monolayers.

Compound	Time (min)	Donor (%)	Receiver (%)
FITC-Dextran (200 µg/mL)	0	100 ± 0.0	0.020 ± 0.001
	15		0.021 ± 0.003
	30		0.024 ± 0.004
	45		0.022 ± 0.002
	60		0.024 ± 0.003
	90		0.023 ± 0.004
	120	93.7 ± 7.8	0.022 ± 0.002
Metoprolol (0.4 mg/mL) +	0	100 ± 0.0	0.021 ± 0.001
FITC-Dextran (200 µg/mL)	15		0.023 ± 0.001
	30		0.020 ± 0.00
	45		0.020 ± 0.00
	60		0.022 ± 0.001
	90		0.023 ± 0.00
	120	94.6 ± 1.4	0.020 ± 0.001

**Table 5:** Adjusted *P_app_* value estimates as a function of drug/concentration, direction, experimental pH, and the cell culture system. BLQ = below the analytical limit of quantification. * Metoprolol (0.4 mg/mL) ± FITC-dextran (200 µg/mL) testing was exclusively performed in canine colonoid monolayers. Values presented herein were aggregated from both metoprolol (0.4 mg/mL) ± FITC-dextran (200 µg/mL) experiments. Transport of metoprolol, atenolol, and propranolol across the Caco-2-derived (human) and canine-colonoid-derived (dog) monolayers. BL→AP and AP→BL transports of the drugs were studied in two different pH conditions, i.e., 6.8 and 7.4 (AP site). Two different monolayers (N = 2) were used for each system for specific directions and pH conditions (for dog: AP→BL, pH 6.8/7.4 or BL→AP, pH 7.4/7.4 or AP→BL, pH 7.4/7.4; for human: AP→BL, pH 6.8/7.4 or BL→AP, pH 7.4/7.4). In total, 20 Caco-2 monolayers and 34 colonoid monolayers were used throughout the study.

Drug	Species	Direction	Well	*P_app_* × 10^−6^	Avg	SD	%CV
Metoprolol (0.4 mg/mL or 584.1 μM)	Human	AP→BL pH 6.8/7.4	1	19.34			
			2	16.45	17.89	2.05	11.43
		BL→AP pH 7.4/7.4	1	13.79			
			2	12.37	13.08	1.01	7.72
	Dog *	AP→BL pH 6.8/7.4	1	8.33			
			2	9.10	8.72	0.55	6.32
		AP→BL pH 7.4/7.4	1	11.19			
			2	9.90	10.54	0.91	8.66
		BL→AP pH 7.4/7.4	1	9.88			
			2	9.94	9.91	0.05	0.46
Metoprolol (0.04 mg/mL or 58.4 μM)	Human	AP→BL pH 6.8/7.4	1	17.54			
			2	13.13	15.33	3.12	20.38
		BL→AP pH 7.4/7.4	1	16.30			
			2	19.03	17.67	1.93	10.92
	Dog	AP→BL pH 6.8/7.4	1	16.83			
			2	18.52	17.68	1.19	6.75
		AP→BL pH 7.4/7.4	1	13.44			
			2	13.50	13.47	0.04	0.32
		BL→AP pH 7.4/7.4	1	11.19			
			2	12.67	11.93	1.04	8.76
Atenolol (0.2 mg/mL or 750.9 μM)	Human	AP→BL pH 6.8/7.4	1	BLQ			
			2	BLQ			
		BL→AP pH 7.4/7.4	1	BLQ			
			2	BLQ			
	Dog	AP→BL pH 6.8/7.4	1	BLQ			
			2	BLQ			
		AP→BL pH 7.4/7.4	1	BLQ			
			2	BLQ			
		BL→AP pH 7.4/7.4	1	BLQ			
			2	BLQ			
Atenolol (2 mg/mL or 7509 μM)	Human	AP→BL pH 6.8/7.4	1	7.25			
			2	8.01	7.63	0.54	7.09
		BL→AP pH 7.4/7.4	1	3.69			
			2	4.97	4.33	0.90	20.89
	Dog	AP→BL pH 6.8/7.4	1	BLQ			
			2	BLQ			
		AP→BL pH 7.4/7.4	1	BLQ			
			2	BLQ			
		BL→AP pH 7.4/7.4	1	BLQ			
			2	BLQ			
Propranolol (1 mg/mL or 3380 μM)	Human	AP→BL pH 6.8/7.4	1	12.12			
			2	12.75	12.44	0.45	3.59
		BL→AP pH 7.4/7.4	1	11.45			
			2	12.43	11.94	0.70	5.84
	Dog	AP→BL pH 6.8/7.4	1	12.23			
			2	11.05	11.64	0.83	7.15
		AP→BL pH 7.4/7.4	1	11.99			
			2	11.97	11.98	0.01	0.09
		BL→AP pH 7.4/7.4	1	10.94			
			2	12.03	11.49	0.77	6.70

**Table 6:** Mean cycle threshold (Ct) for each of the evaluated genes in canine colonoid- or Caco-2-derived monolayers.

Species	Category	Gene Full Name	Symbol	Cycle Threshold (Ct)	
				Mean	SD
*Canis lupus familiaris* (dog) (colonoid-derived monolayer)	Housekeeping gene	Glyceraldehyde-3-phosphate dehydrogenase	GAPDH	20.67	1.52
	Tight and adherens junction proteins	Cadherin 1	CDH1	24.49	0.12
		Occludin	OCLN	25.92	0.12
		Tight junction protein 1	TJP1	29.80	0.15
	Intestinal epithelial cell differentiation markers	Mucin 2	MUC2	25.83	0.22
		Neurogenin 3	NEUROG3	33.75	0.37
		Intestinal alkaline phosphatase	ALP	26.91	0.76
	Stem cell markers	Olfactomedin 4	OLFM4	22.19	0.91
		HOP homeobox	HOPX	25.85	1.01
		Prominin 1	PROM1	26.48	0.10
		SRY-box transcription factor 9	SOX9	26.27	0.12
		Leucine-rich repeat-containing G-protein-coupled receptor 5	LGR5	30.38	0.68
	Transporters	Organic anion transporting polypeptide	Oatp2b1	32.41	0.28
			Oatp2b1 (liver tissue) (used as positive control)	21.41	0.07
		Multidrug resistance p-glycoprotein	Mdr1	25.36	0.14
	Cytochrome P450 (CYP) enzymes	Cytochrome P-450 3a12	Cyp3a12	27.40	0.70
		Cytochrome P450 2b11	Cyp2b11	30.48	1.29
		Cytochrome P450 2C21	Cyp2c21 (Cyp2c18)	31.24	0.08
*Homo sapiens* (human) (Caco-2-derived monolayer)	Housekeeping gene	Glyceraldehyde-3-phosphate dehydrogenase	GAPDH	20.21	0.08
	Transporters	Organic anion transporting polypeptide 2B1/solute carrier organic anion transporter family member 2B1	OATP2B1 (SLCO2B1)	28.11	0.10
		ATP binding cassette subfamily B member 1	ABCB1 (MDR1)	35.53	1.76
	Cytochrome P450 (CYP) enzymes	Cytochrome P450 family 3 subfamily A member 4	CYP3A4	32.39	0.22
		Cytochrome P450 family 2 subfamily B member 6	CYP2B6	33.22	0.30
		Cytochrome P450 family 2 subfamily C member 9	CYP2C9	31.34	1.46
		Cytochrome P450 family 2 subfamily C member 19	CYP2C19	38.34	0.06

Values are expressed as the arithmetic mean of the data and one standard deviation of N = 3 monolayers.

## Data Availability

All data generated or analyzed during this study are included in this published article (and its Supplementary Information files).

## Supplementary Materials

The following Supporting Information can be downloaded at: https://www.mdpi.com/article/10.3390/cells12091269/s1: Figure S1: Representative calibration curves at pH 6.8 for A. Atenolol, B. Metoprolol, C. Propranolol and at pH 7.4 for D. Atenolol, E. Metoprolol, and F. Propranolol; Table S1A: Continuous TEER (Ω×cm^2^) analysis over 15 days was used to assess the integrity of each colonoid monolayer (N = 18 used for each drug study) (canine colonoid-derived monolayers with IDs Colonoid#M1 to Colonoid#M54 were used); Table S1B: Continuous TEER (Ω×cm^2^) analysis of the Caco-2 cell monolayer (N = 12 used for each drug study) over 23 days was used to assess monolayer integrity (Caco-2-derived monolayers with IDs Caco-2#M1 to Caco-2#M36 were used); Table S2A: Estimation of metoprolol (ppm) absorptive (AB) and secretive permeability (BA) in canine colonoid-derived (dog) and Caco-2-derived (human) monolayers. *Metoprolol (0.4 mg/mL) +/− FITC Dextran (200 µg/mL) testing was exclusively performed in canine colonoid monolayers; Table S2B: Estimation of atenolol (ppm) absorptive (AB) and secretive permeability (BA) in canine colonoid-derived (dog) and Caco-2-derived (human) monolayers; Table S2C: Estimation of propranolol (ppm) absorptive (AB) and secretive permeability (BA) in canine colonoid-derived (dog) and Caco-2-derived (human) monolayers.
