# Supplementary material for: Canine Intestinal Organoids as a Novel In Vitro Model of Intestinal Drug Permeability: A Proof-of-Concept Study
Source: Cells. 2023 Apr 27;12(9):1269. doi: 10.3390/cells12091269 (PMC10177590; doi:10.3390/cells12091269)
Supplement: Supplementary file 1 [file cells-12-01269-s001.zip › cells-2349741-supplementary.pdf]

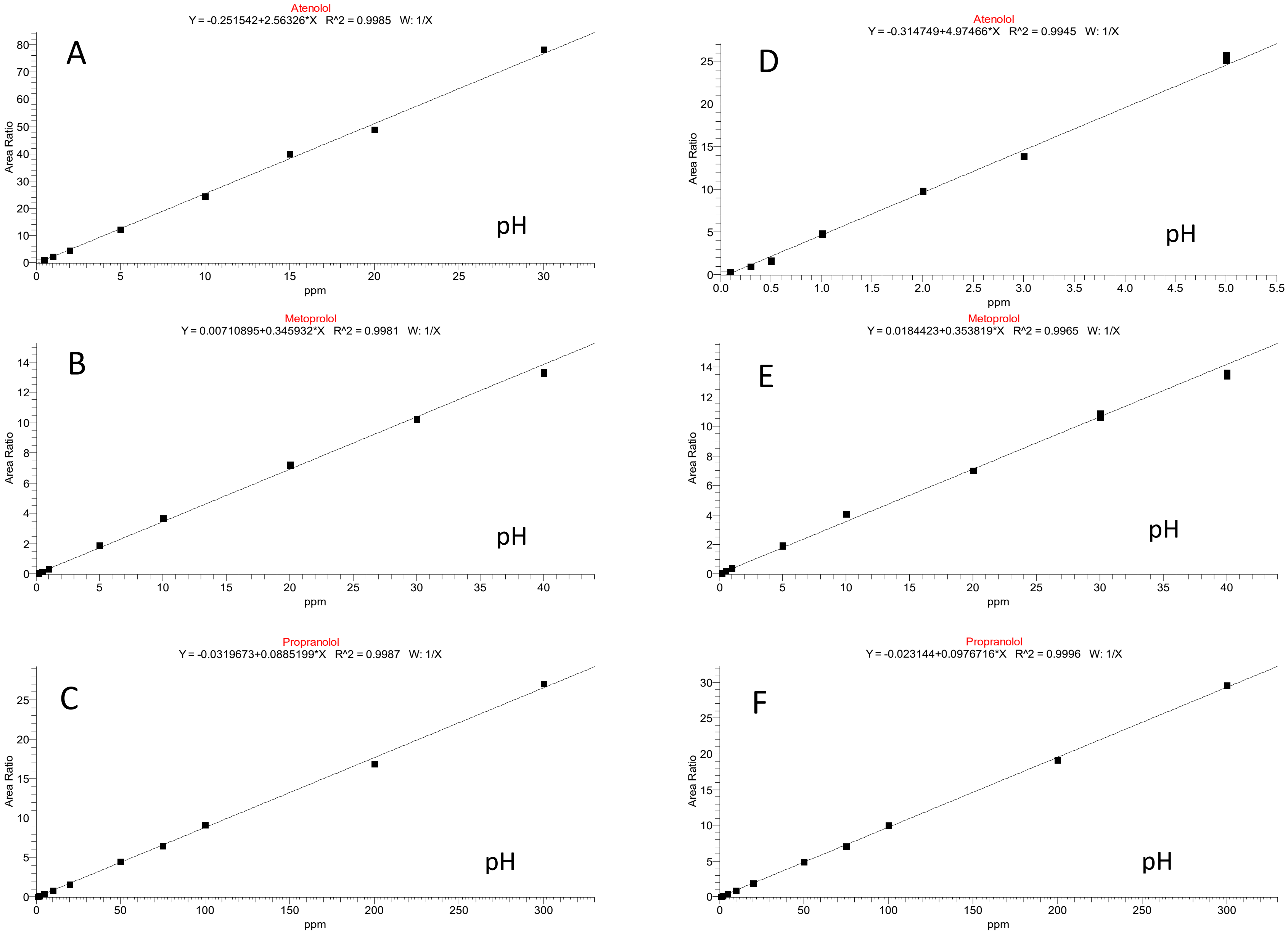

Atenolol
Y = -0.251542+2.56326*X   R^2 = 0.9985   W: 1/X
A
pH
Area Ratio
ppm
Atenolol
Y = -0.314749+4.97466*X   R^2 = 0.9945   W: 1/X
D
pH
Area Ratio
ppm
Metoprolol
Y = 0.00710895+0.345932*X   R^2 = 0.9981   W: 1/X
B
pH
Area Ratio
ppm
Metoprolol
Y = 0.0184423+0.353819*X   R^2 = 0.9965   W: 1/X
E
pH
Area Ratio
ppm
Propranolol
Y = -0.0319673+0.0885199*X   R^2 = 0.9987   W: 1/X
C
pH
Area Ratio
ppm
Propranolol
Y = -0.023144+0.0976716*X   R^2 = 0.9996   W: 1/X
F
pH
Area Ratio
ppm

Supplemental Figure S1: Representative calibration curves at pH 6.8 for A. Atenolol, B. Metoprolol, C. Propranolol and at pH 7.4 for D. Atenolol, E. Metoprolol, and F. Propranolol.

Supplemental Table S1

	Days	Metoprolol		Atenolol		Propranolol	
Canine colonoid monolayer		Mean	SD	Mean	SD	Mean	SD
	1	13.26	3.30	18.03	5.40	10.58	2.91
	2	79.19	23.40	83.54	22.71	71.83	15.82
	3	512.42	200.30	547.54	200.73	441.38	209.88
	4	1152.00	102.40	1033.59	201.67	1270.17	117.52
	5	1946.83	219.80	1843.28	118.78	1665.94	148.29
	6	2444.75	107.50	2232.74	126.62	2529.14	172.05
	7	2724.61	127.39	2924.34	166.15	2605.07	130.83
	8	3033.20	100.71	3217.01	275.67	3136.60	237.59
	9	3595.95	100.51	3659.72	121.17	3711.78	112.31
	10	4006.24	250.44	4211.72	251.65	4223.69	224.59
	11	4099.73	357.43	4344.74	358.71	4238.37	253.15
	12	3977.80	341.96	4205.32	342.46	4260.19	449.26
	13	3032.24	356.66	3367.85	96.98	3402.98	87.70
	14	2541.86	315.42	2842.84	310.04	2703.76	211.70
	15	1796.67	196.58	2121.39	76.46	1963.67	156.75

Supplemental Table S1A. Continuous TEER (Ω×cm^2) analysis over 15 days was used to assess the integrity of each colonoid monolayer (n = 18 used for each drug study) (canine colonoid-derived monolayers with IDs Colonoid#M1 to Colonoid#M54 were used).

	Days	Metoprolol		Atenolol		Propranolol	
**Caco-2 monolayer**		**Mean**	**SD**	**Mean**	**SD**	**Mean**	**SD**
	**1**	34.90	10.49	28.45	5.44	31.56	3.55
	**3**	178.04	30.09	161.35	23.92	172.69	28.43
	**4**	239.66	12.36	244.60	18.88	228.29	8.71
	**6**	280.12	28.32	265.91	29.47	253.44	20.01
	**8**	278.24	21.32	288.21	22.82	273.78	10.78
	**10**	314.23	31.73	300.84	39.24	308.06	27.59
	**12**	346.40	33.54	347.61	40.89	334.55	34.06
	**14**	370.95	49.17	367.37	39.87	360.10	37.79
	**16**	412.15	43.16	405.29	41.85	395.06	26.74
	**18**	487.34	61.27	495.66	58.57	472.16	66.74
	**19**	474.62	71.22	523.15	36.54	513.53	35.96
	**20**	526.16	43.03	503.88	41.64	506.66	46.00
	**21**	502.34	42.66	493.98	34.02	483.53	34.80
	**22**	322.55	45.85	327.06	71.77	314.02	74.84
	**23**	259.27	68.46	251.71	53.37	241.01	49.93

Supplemental Table S1B. Continuous TEER (Ω×cm^2) analysis of the Caco-2 cell monolayer (n = 12 used for each drug study) over 23 days was used to assess monolayer integrity (Caco-2-derived monolayers with IDs Caco-2#M1 to Caco-2#M36 were used).

Supplemental Table S2

Drug Conc. (mg/mL)	Species	AP (pH)	Direction	Time (min)	Don. Conc. (ppm)	Rec. Conc. (ppm)	Replicates
Metoprolol (0.4mg/mL)	Human	6.8	A-B	0	487	None Detected	1
				0	455	None Detected	2
				120	396	13.7	1
				120	410	12.6	2
				15		2.86	1
				15		2.43	2
				30		4.91	1
				30		4.42	2
				45		5.75	1
				45		7.68	2
				60		7.54	1
				60		7.02	2
				90		10.9	1
				90		9.03	2
Metoprolol (0.4mg/mL)	Human	7.4	B-A	0	472	None Detected	1
				0	488	None Detected	2
				120	452	22.7	1
				120	499	22.8	2
				15		8.41	1
				15		8.40	2
				30		10.9	1

				30		12.9	2
				45		13.3	1
				45		13.4	2
				60		14.5	1
				60		13.3	2
				90		18.1	1
				90		16.7	2
Metoprolol (0.4mg/mL)	Dog	6.8	A-B	0	231	None Detected	1
				0	229	None Detected	2
				120	197	5.09	1
				120	194	4.33	2
				15		1.70	1
				15		2.01	2
				30		2.48	1
				30		2.50	2
				45		3.34	1
				45		3.15	2
				60		3.80	1
				60		3.78	2
				90		4.34	1
				90		4.49	2
Metoprolol (0.4mg/mL)	Dog	7.4	A-B	0	235	None Detected	1
				0	231	None Detected	2
				120	168	6.49	1
				120	186	5.83	2
				15		2.44	1

				15		1.99	2
				30		3.38	1
				30		2.69	2
				45		3.90	1
				45		3.50	2
				60		4.80	1
				60		4.44	2
				90		5.24	1
				90		5.14	2
Metoprolol (0.4mg/mL)	Dog	7.4	B-A	0	237	None Detected	1
				0	237	None Detected	2
				120	223	10.3	1
				120	215	9.90	2
				15		8.11	1
				15		8.02	2
				30		8.40	1
				30		8.34	2
				45		9.08	1
				45		8.70	2
				60		9.40	1
				60		8.69	2
				90		9.24	1
				90		9.51	2
Metoprolol (0.04 mg/mL)	Human	6.8	A-B	0	48.5	None Detected	1
				0	52.6	None Detected	2
				120	42.1	1.43	1

				120	43.3	1.62	2
				15		0.310	1
				15		0.273	2
				30		0.356	1
				30		0.465	2
				45		0.806	1
				45		None Detected	2
				60		0.695	1
				60		0.809	2
				90		1.62	1
				90		1.19	2
Metoprolol (0.04 mg/mL)	Human	7.4	B-A	0	53.5	None Detected	1
				0	54.8	None Detected	2
				120	50.7	3.00	1
				120	46.3	3.28	2
				15		0.879	1
				15		0.806	2
				30		1.16	1
				30		1.27	2
				45		1.64	1
				45		1.79	2
				60		1.75	1
				60		1.65	2
				90		2.25	1
				90		2.32	2

Metoprolol (0.04 mg/mL)	Dog	6.8	A-B	0	23.6	None Detected	1
				0	12.4	None Detected	2
				120	11.2	0.546	1
				120	26.2	0.433	2
				15		< 0.2	1
				15		< 0.2	2
				30		< 0.2	1
				30		< 0.2	2
				45		0.292	1
				45		< 0.2	2
				60		0.381	1
				60		0.302	2
				90		0.458	1
				90		0.342	2
Metoprolol (0.04 mg/mL)	Dog	7.4	A-B	0	24.3	None Detected	1
				0	24.7	None Detected	2
				120	17.4	0.642	1
				120	17.1	0.647	2
				15		< 0.2	1
				15		< 0.2	2
				30		0.291	1
				30		0.278	2
				45		0.351	1
				45		0.384	2
				60		0.488	1

				60		0.458	2
				90		0.549	1
				90		0.506	2
Metoprolol (0.04 mg/mL)	Dog	7.4	B-A	0	23.1	None Detected	1
				0	24.7	None Detected	2
				120	23	1.09	1
				120	22.2	1.05	2
				15		0.696	1
				15		0.638	2
				30		0.801	1
				30		0.731	2
				45		0.844	1
				45		0.844	2
				60		0.896	1
				60		0.888	2
				90		0.963	1
				90		0.975	2
Metoprolol (0.4mg/mL)	Dog	6.8	A-B	0	281	None Detected	1
				0	287	None Detected	2
				120	260	2.94	1
				120	247	2.60	2
				15		0.293	1
				15		0.200	2
				30		0.593	1
				30		0.814	2
				45		1.02	1

				45		0.819	2
				60		1.49	1
				60		0.888	2
				90		2.70	1
				90		1.67	2
Metoprolol (0.4mg/mL) + FITC Dextran (200 µg/mL)	Dog	6.8	A-B	0	283	None Detected	1
				0	287	None Detected	2
				120	252	3.03	1
				120	267	3.20	2
				15		0.368	1
				15		0.282	2
				30		0.897	1
				30		0.868	2
				45		1.18	1
				45		1.12	2
				60		1.54	1
				60		1.51	2
				90		2.60	1
				90		2.61	2

Supplemental Table S2A: Estimation of metoprolol (ppm) for absorptive (AB) and secretive permeability (BA) in canine colonoid-derived (dog) and Caco-2-derived (human) monolayers. *Metoprolol (0.4 mg/mL) +/- FITC Dextran (200 µg/mL) testing was exclusively performed in canine colonoid monolayers.

Drug Conc. (mg/mL)	Species	AP (pH)	Direction	Time (min)	Don. Conc. (ppm)	Rec. Conc. (ppm)	Replicates
Atenolol (2 mg/mL)	Human	6.8	A-B	0	3772.0	None Detected	1
				0	3890.0	None Detected	2
				120	2895.0	52.2	1
				120	2650.0	50.8	2
				15		2.50	1
				15		1.3	2
				30		4.20	1
				30		2.1	2
				45		11.4	1
				45		7.4	2
				60		18.1	1
				60		9.0	2
				90		44.8	1
				90		45.4	2
Atenolol (2 mg/mL)	Human	7.4	B-A	0	902.0	None Detected	1
				0	1090.0	None Detected	2
				120	1053.0	8.8	1
				120	989.0	9.6	2
				15		1.9	1
				15		1.9	2
				30		2.5	1
				30		2.6	2
				45		4.3	1
				45		4.40	2

				60		4.3	1
				60		4.2	2
				90		10.9	1
				90		11.0	2
Atenolol (2 mg/mL)	Dog	6.8	A-B	0	993	None Detected	1
				0	1031	None Detected	2
				120	1073	< 0.5	1
				120	1019	< 0.5	2
				15		< 0.5	1
				15		< 0.5	2
				30		< 0.5	1
				30		< 0.5	2
				45		< 0.5	1
				45		< 0.5	2
				60		< 0.5	1
				60		< 0.5	2
				90		< 0.5	1
				90		< 0.5	2
Atenolol (2 mg/mL)	Dog	7.4	A-B	0	544	None Detected	1
				0	547	None Detected	2
				120	438	< 0.5	1
				120	498	< 0.5	2
				15		< 0.5	1
				15		< 0.5	2
				30		< 0.5	1
				30		< 0.5	2

				45		< 0.5	1
				45		< 0.5	2
				60		< 0.5	1
				60		< 0.5	2
				90		< 0.5	1
				90		< 0.5	2
Atenolol (2 mg/mL)	Dog	7.4	B-A	0	522	None Detected	1
				0	501	None Detected	2
				120	485	< 0.5	1
				120	541	< 0.5	2
				15		< 0.5	1
				15		< 0.5	2
				30		< 0.5	1
				30		< 0.5	2
				45		< 0.5	1
				45		< 0.5	2
				60		< 0.5	1
				60		< 0.5	2
				90		< 0.5	1
				90		< 0.5	2
Atenolol (2 mg/mL)	Human	6.8	A-B	0	266.0	None Detected	1
				0	249.0	None Detected	2
				120	266.0	<0.5	1
				120	265.0	0.62	2
				15		<0.5	1
				15		<0.5	2

				30		<0.5	1
				30		<0.5	2
				45		<0.5	1
				45		<0.5	2
				60		<0.5	1
				60		<0.5	2
				90		<0.5	1
				90		<0.5	2
Atenolol (2 mg/mL)	Human	7.4	B-A	0	173.0	None Detected	1
				0	157.0	None Detected	2
				120	158.0	0.77	1
				120	162.0	1.79	2
				15		<0.5	1
				15		<0.5	2
				30		<0.5	1
				30		<0.5	2
				45		<0.5	1
				45		<0.5	2
				60		<0.5	1
				60		<0.5	2
				90		0.68	1
				90		0.84	2
Atenolol (2 mg/mL)	Dog	6.8	A-B	0	274.9	<0.5	1
				0	274.1	<0.5	2
				120	273.2	<0.5	1
				120	275.0	<0.5	2

				15		<0.5	1
				15		<0.5	2
				30		<0.5	1
				30		<0.5	2
				45		<0.5	1
				45		<0.5	2
				60		<0.5	1
				60		<0.5	2
				90		<0.5	1
				90		<0.5	2
Atenolol (2 mg/mL)	Dog	7.4	A-B	0	256.6	None Detected	1
				0	241.1	None Detected	2
				120	249.4	<0.5	1
				120	238.2	<0.5	2
				15		<0.5	1
				15		<0.5	2
				30		<0.5	1
				30		<0.5	2
				45		<0.5	1
				45		<0.5	2
				60		<0.5	1
				60		<0.5	2
				90		<0.5	1
				90		<0.5	2
Atenolol (2 mg/mL)	Dog	7.4	B-A	0	259.0	<0.5	1
				0	262.5	<0.5	2

				120	261.8	2	1
				120	258.6	Approximately 0.7	2
				15		<0.5	1
				15		<0.5	2
				30		<0.5	1
				30		<0.5	2
				45		<0.5	1
				45		<0.5	2
				60		Approximately 0.7	1
				60		<0.5	2
				90		<0.5	1
				90		<0.5	2

Supplemental Table S2B: Estimation of atenolol (ppm) for absorptive (AB) and secretive permeability (BA) in canine colonoid-derived (dog) and Caco-2-derived (human) monolayers.

Drug Conc. (mg/mL)	Species	AP (pH)	Direction	Time (min)	Don. Conc. (ppm)	Rec. Conc. (ppm)	Replicates
Propranolol (1 mg/mL)	Human	6.8	A-B	0	588	None Detected	1
				0	612	None Detected	2
				120	455	18.6	1
				120	489	17.7	2
				15		3.01	1
				15		2.65	2
				30		7.13	1
				30		5.09	2
				45		8.23	1
				45		8.61	2
				60		8.98	1
				60		10.4	2
				90		11.8	1
				90		15.6	2
Propranolol (1 mg/mL)	Human	7.4	A-B	0	598	None Detected	1
				0	585	None Detected	2
				120	421	14.9	1
				120	451	15.6	2
				15		2.66	1
				15		1.58	2
				30		4.33	1
				30		4.55	2

				45		6.04	1
				45		7.05	2
				60		8.37	1
				60		8.63	2
				90		10.6	1
				90		13.6	2
Propranolol (1 mg/mL)	Dog	6.8	A-B	0	1186	None Detected	1
				0	1164	None Detected	2
				120	882	35.0	1
				120	912	33.0	2
				15		11.0	1
				15		12.0	2
				30		18.4	1
				30		17.0	2
				45		22.6	1
				45		21.0	2
				60		28.5	1
				60		26.3	2
				90		30.2	1
				90		28.5	2
Propranolol (1 mg/mL)	Dog	7.4	A-B	0	1175	None Detected	1
				0	1080	None Detected	2
				120	817	33.0	1
				120	851	31.5	2

				15		10.7	1
				15		11.0	2
				30		16.4	1
				30		17.7	2
				45		22.9	1
				45		21.1	2
				60		24.8	1
				60		27.7	2
				90		26.7	1
				90		31.0	2
Propranolol (1 mg/mL)	Dog	7.4	B-A	0	1110	None Detected	1
				0	1121	None Detected	2
				120	914	44.3	1
				120	979	47.2	2
				15		27.6	1
				15		26.5	2
				30		33.9	1
				30		36.7	2
				45		41.4	1
				45		44.5	2
				60		32.7	1
				60		42.5	2
				90		38.0	1
				90		43.5	2

Supplemental Table S2C: Estimation of propranolol (ppm) absorptive (AB) and secretive permeability (BA) in canine colonoid-derived (dog) and Caco-2-derived (human) monolayers.
